# The effect of mutational robustness on the evolvability of multicellular organisms and eukaryotic cells

**DOI:** 10.1111/jeb.14180

**Published:** 2023-05-31

**Authors:** Pengyao Jiang, Martin Kreitman, John Reinitz

**Affiliations:** 1 Department of Ecology & Evolution, University of Chicago, Chicago, Illinois, USA; 2 Department of Genome Sciences, University of Washington, Seattle, Washington, USA; 3 Institute for Genomics & Systems Biology, Chicago, Illinois, USA; 4 Department of Statistics, University of Chicago, Chicago, Illinois, USA; 5 Department of Molecular Genetics and Cell Biology, University of Chicago, Chicago, Illinois, USA

**Keywords:** adaptation, Boolean model, canalization, eukaryotes, hierarchical phenotype, neural net, population simulations, Wright–Fisher

## Abstract

Canalization involves mutational robustness, the lack of phenotypic change as a result of genetic mutations. Given the large divergence in phenotype across species, understanding the relationship between high robustness and evolvability has been of interest to both theorists and experimentalists. Although canalization was originally proposed in the context of multicellular organisms, the effect of multicellularity and other classes of hierarchical organization on evolvability has not been considered by theoreticians. We address this issue using a Boolean population model with explicit representation of an environment in which individuals with explicit genotype and a hierarchical phenotype representing multicellularity evolve. Robustness is described by a single real number between zero and one which emerges from the genotype–phenotype map. We find that high robustness is favoured in constant environments, and lower robustness is favoured after environmental change. Multicellularity and hierarchical organization severely constrain robustness: peak evolvability occurs at an absolute level of robustness of about 0.99 compared with values of about 0.5 in a classical neutral network model. These constraints result in a sharp peak of evolvability in which the maximum is set by the fact that the fixation of adaptive mutations becomes more improbable as robustness decreases. When robustness is put under genetic control, robustness levels leading to maximum evolvability are selected for, but maximal relative fitness appears to require recombination.

## 1 | INTRODUCTION

Phenotypes display a spectrum of sensitivities to environmental cues. At one extreme are highly plastic traits, for which specific environmental or physiological signals induce alternative optimal phenotypes. At the other extreme are invariant, that is, canalized or robust, traits for which the influence of the external environment has been attenuated. Natural selection can establish setpoints along this spectrum of environmental sensitivity to optimize populations to specific environmental regimes (Ancel, 1999; Ehrenreich & Pfennig, 2015; Palacio-López et al., 2015; Stewart et al., 2012).

Here, we address questions concerning phenotypes that fall at one end of the spectrum—canalized traits—and how they evolve. First brought to our attention by Waddington (1942), canalization involves not only the attenuation of environmental variability, but also insensitivity to genetic mutation and developmental noise. Canalization is widely recognized as a general feature in developmental processes, due both to the structural properties of developmental networks as well as to the evolution of traits governed by stabilizing selection (Barkai & Leibler, 1997; Félix & Barkoulas, 2015; Meiklejohn & Hartl, 2002; Siegal & Bergman, 2002). Recent studies investigating gene regulatory networks have also found support for Waddington’s classical idea of canalization. It has been shown that gene expression is buffered against perturbations by interactions between genes in *trans* that give rise to attractor states in the epigenetic landscape (Huang, 2012; Manu et al., 2009a, 2009b; Moris et al., 2016). Similarly, gene expression is buffered by interactions in *cis* within genes because the large noncoding regulatory regions buffer the effect of single base changes (Barr et al., 2019). Canalization in *cis* is a form of ‘concentration’, in the mathematical sense. Concentration occurs when a mathematical function of many random variables with large variance has a small variance (cf. Talagrand [1995], Gromov [2007], Boucheron et al. [2013]). In the evolutionary context, the key property of canalization is its effect on reducing or suppressing the phenotypic consequences of mutation, a property frequently referred to as ‘genetic robustness’.

The high reproducibility of a strongly canalized trait in the presence of genetic robustness attenuates heritable phenotypic variation, thus reducing the rate of adaptation (Fisher, 1930). Yet, canalized traits evolve adaptively over phylogenetic time. Scutellar bristle number in *Drosophila*, for example, is not only highly canalized (Rendel, 1959; Wheeler, 1987), but also differs characteristically within family Drosophilidae (Wheeler, 1987), making it a useful taxonomic feature in species classification. Under what circumstances, then, can canalized traits evolve? Early conceptual models invoke gene–environmental interactions upon specific environmental stimuli to release phenotypic variation on which natural selection can act (Simpson, 1953; Waddington, 1942, 1953). This process involves environmental factors interacting with existing genetic variation to create novel phenotypes (Gibson & Dworkin, 2004; Gibson & Hogness, 1996; Scharloo, 1991).

Mutation can also release otherwise suppressed phenotypic variation. The number of vibrissae is typically 19 in the house mouse, but in *Tabby* mutants, this number can vary (Dun & Fraser, 1958). Similarly in *Drosophila*, the number of scutellar bristles varies in a *scute* mutant but not wild type (Rendel, 1965). Release of phenotypic variation is not restricted to mutation in genetic components regulating a specific phenotype. There may also be genetic factors that act as global regulators of robustness, sometimes called ‘evolutionary capacitors’. Rutherford and Lindquist (1998) proposed that Hsp90, a chaperone involved in protein folding, is one such factor, buffering phenotypic variation under non-stressed environmental conditions, but releasing this variation at opportune times in response to specific environmental stressors (Gibson, 2009; Masel, 2005; Masel & Siegal, 2009; Paaby & Rockman, 2014). Additional regulators have been found in a genetic screen in yeast (Levy & Siegal, 2008). But evidence for the existence of genetic capacitors that contribute to adaptation remains under debate (Levy & Siegal, 2008; Masel, 2005; Masel & Siegal, 2009; Meiklejohn & Hartl, 2002). The strongest support for this hypothesis comes from a study that found directionality of eye size change upon Hsp90 induction in the cavefish, a phenotype that is otherwise stabilized by Hsp90 in the ancestral surface populations (Rohner et al., 2013).

The largest gap in our understanding of how canalized traits evolve, however, does not revolve around uncertainly about the specific molecular mechanisms of canalization that contribute to adaptation, such as Hsp90. Rather, it revolves around theoretical issues: the degree of robustness that can be maintained in the population under stabilizing selection, the extent to which a lowering of robustness is required when adapting to a novel environment and finally because robustness is itself a property of phenotype, the representation of phenotype in theoretical models becomes an issue as well. Below, we summarize key findings about robustness against mutation.

In contrast to the intuitive appeal of the idea that release of cryptic variation through reduced robustness facilitates adaptation, A. Wagner has argued that robustness can facilitate adaptation (Wagner, 2008, 2012). The central idea is that of a genotype network (originally referred to as a ‘neutral network’), in which the measure of evolvability is the accessibility of new phenotypes from all genotypes connected by phenotypically neutral mutations. In these studies, the picture of the genotype–phenotype map was inspired by the secondary structure of RNA as determined from its sequence. Experimental support for this picture has been adduced from studies of both RNA (Hayden et al., 2011) and protein (Bloom et al., 2006) structure and function.

These ideas were formulated in a mathematically precise manner in a study that abstracted the key features of a neutral network into a population genetics picture in which the statistical properties of the fitness landscape could be described by a small number of parameters (Draghi et al., 2010). A key idea is that one optimal phenotype is maintained in the population; in a given environment, all alternative phenotypes are assumed to be lethal or highly deleterious. Robustness is the probability that a neighbouring genotype will be neutral. In the face of a new environment, defined by the authors as the random selection of a new optimal phenotype, adaptation is achieved by positive selection acting on new mutant genotypes that are one step away from particular (i.e. not all) genotypes in the neutral network, thus providing epistasis between new mutations and pre-existing alleles in the neutral network to create novel phenotypes. Evolvability here is defined in terms of the expected waiting time for an adaptive mutation considered as a function of robustness, which can be varied at will in the model. A major strength of this analysis is that by imposing temporal continuity on the evolutionary process, the authors obtain a Fokker–Planck equation with exact solutions, from which the expected adaptation time can be obtained in closed form.

Draghi et al. (2010) showed that if all phenotypes were accessible by a single mutation, evolvability was indeed a monotonically decreasing function of robustness, but that in the more realistic case where some novel phenotypes could only be reached by multiple mutations, evolvability was maximized at intermediate robustness levels. The authors quantified robustness by q, the probability that a mutation was phenotypically neutral. The generic finding was that evolvability was maximized over intermediate values of q, with the exact maximum dependent on neutral network parameters. In all cases, the authors found a broad area of evolvability with sharp decreases at the limits of extremely high and extremely low values of q. At q=1, complete robustness, evolvability is zero because no new phenotypes are accessible. As q decreases from unity, new phenotypes begin to be accessible from the neutral network and evolvability increases. As q→0, all mutations express an alternative phenotype and the neutral network disappears. These analytic results were well supported by numerical simulations of both the underlying master equation model and on simulated RNA structures.

These results do not address the effects of multicellularity and the hierarchical organization of eukaryotic cells on robustness and adaptation. Most of the early experimental work on robustness and canalization cited above was carried out in multicellular animals. Metazoan organisms undergo development, a process that unfolds in time during which cells come to express different sets of genes in a precise spatial pattern. Although the relationship between development and evolution is now a subject of active comparative studies among molecular geneticists, evo-devo has never been theoretically unified with population genetics. The same theoretical issues extend to the evolutionary effects of the hierarchical structure of eukaryotic cells. This theoretical problem arises from the fact that population genetics classically treats fitness as a direct function of genotype, with epistasis occurring as a correction factor to additive contributions from different genetic loci. Studies of canalization and evolvability based on population genetics (Carter et al., 2005; Draghi et al., 2010; Eshel & Matessi, 1998; Hermisson & Wagner, 2004; Wagner et al., 1997) do not explicitly consider development, which is fundamentally epistatic in nature. Epistatic models used for developmental problems (Mjolsness et al., 1991; Reinitz et al., 1995; Reinitz & Sharp, 1995) have been applied to evolutionary problems for some time (Draghi & Whitlock, 2012; Siegal & Bergman, 2002; Wagner, 1994, 1996), but only in the context of considering the state of a single cell. An advantage of such approaches is that they permit analyses that can address a structurally realistic picture that represents metabolic and/or genetic networks under the control of an explicitly represented genome that can respond to the environment (Crombach & Hogeweg, 2008; Cuypers et al., 2017), or molecularly realistic models of a single locus whose evolvability is controlled by a second locus with anti-terminator activity (Griswold & Masel, 2009). None of these studies address the effects of the temporal nature of development or the requirement for multiple cellular states in a metazoan organism, although it has been suggested that these effects are pervasive on the entire history of metazoan evolution (Buss, 1987).

In this work, we address this problem by analysing the relationship between robustness and evolvability with a theoretical model that contains an explicit representation of differing cell types arising from differential gene expression in different cells of a metazoan organism. The model’s representation of cell types can also be thought of as applying to the organelles of eukaryotic cells and other hierarchical phenotypes, although our primary motivation was to address the better characterized issue of multicellularity. The model employs an explicit genotype to phenotype map, treats fitness as arising from interactions between the phenotype and environment and has tunable control of robustness. For simplicity, genotypes and phenotypes are encoded using Boolean vectors. The widely different timescales of ontogeny and evolution raise serious computational problems; for that reason, we do not consider the temporal aspects of development. Our model (1) draws on established principles of gene regulatory control of cell types, each of which constitutes a trait (we hypothesize gene regulatory networks that determine trait values); (2) specifies a genotype to phenotype map that represents pleiotropy (multiple genotypes map to multiple cell types) that is (3) calculated from alleles (ON/OFF) and the regulatory activities of the proteins they produce (activating/repressing); (4) has robustness parameters that control the sensitivity of each mutation; (5) allows mutational inputs to the robustness parameter, enabling (6) dynamical analysis of the evolution of both traits and robustness simultaneously via a standard Wright–Fisher population genetic model with selection. This dynamical formulation permits us to evaluate evolvability by comparing the mean fitness of populations under different evolutionary conditions. Our specific mathematical choices, to be described below, are intended to produce the simplest possible model that incorporates features 1 to 6 above. We employ the model to explore the relationship between mutational robustness and evolvability for populations at the phenotypic optimum and for populations that are challenged to evolve towards a novel phenotypic optimum. A general theme of the results presented below is that when genes control a complex hierarchical phenotype, adaptive changes in some selectable traits tend to be accompanied by fitness changes in other traits controlled by the same gene, forcing adaptation to occur at a much higher level of robustness than would be true otherwise.

## 2 | THE MODEL

The model equations presented below are motivated by some basic concepts of developmental and evolutionary genetics. Metazoan organisms are composed of cells, each with the same genotype but with each cell type expressing a different set of genes. The cell type of each differentiated cell can be considered as phenotype at a cellular level. Phenotype at the organismal level depends on the entire collection of differentiated cell types. Selection occurs at the organismal level, and depends on both organismal phenotype and environment. The model equations below are a minimal representation of this two-level hierarchical phenotype, with the organismal fitness dependent on the presence of a necessary set of cell types.

Both cellular and organismal phenotype are under genetic control, each by a different set of genes. In developmental genetics, genes can be roughly classified as ‘selectors’ or ‘effectors’. Selector genes (also called ‘master regulator genes’) control organismal phenotype by altering the number and spatial pattern of cell types. Such genes are often involved in signalling or transcriptional regulation, with the BMP (Montanari et al., 2022) and HOX-complex (Pick & Heffer, 2012) genes, respectively, representing examples of each class. Selector genes tend to have homologues throughout metazoa, but are not found in single celled organisms. By contrast, effector genes (also called ‘terminal differentiation genes’) produce products—actins, pigments, metabolic enzymes, ion channels and so on—that implement a particular cell type. Effector genes tend to have homologues in both metazoan and single-celled organisms. The model equations below represent control of effector genes by selector genes with a minimal regulatory network.

The necessity for multiple cell types and the distinction between selector and effector genes are the central motivations for our model equations. We imagine effector genes to evolve sufficiently slowly compared to selector genes that our model’s genotype–phenotype map will describe selector genes evolving and producing changing phenotypes by acting on a relatively fixed network of effector genes. Phenotype is specified by a collection of cell types, with the fitness of a given phenotype defined by its interaction with the surrounding environment.

**FIGURE 1:**
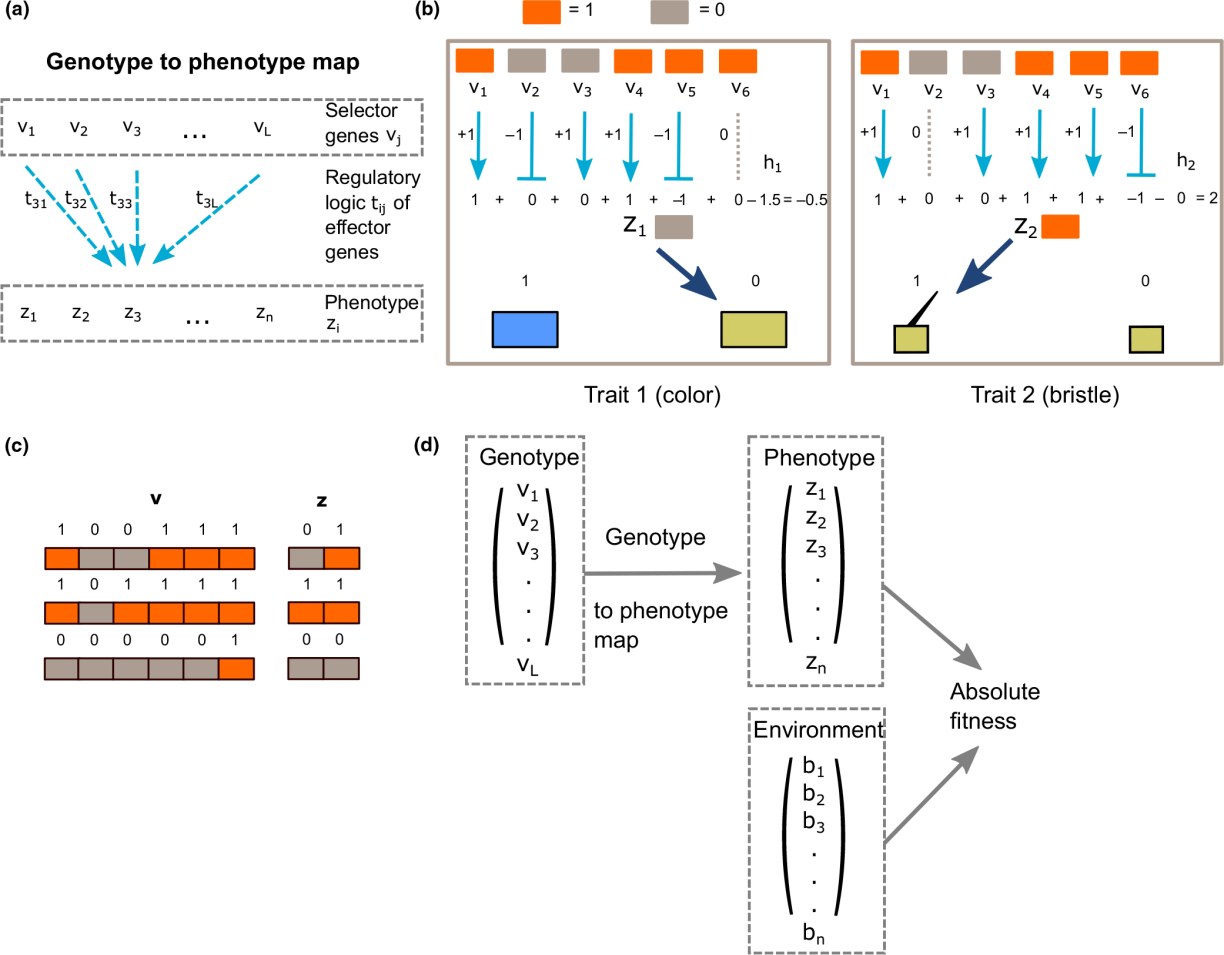
Overview of the model. (a) The genotype–phenotype map. Selector genes $v_{j}$ act on an effector gene regulatory network $t_{ij}$ to produce a traits in the form of cell types $z_{i}$. (b) Schematic example of two cell types and hence traits determined by the genotype to phenotype map within one individual, from Equation (1). $z_{1}$ determines colour, and $z_{2}$ determines the presence or absence of a bristle. In this example, the number of genes regulating the phenotype is 6, and γ is set to 1. Actions of each gene on each phenotype are marked by arrows. Activation is represented by arrows with a triangle end, while repression is represented by a bar end. A grey dashed line means no effect. Under the state of v shown, $z_{1}=0$ and $z_{2}=1$. (c) Examples of different genotype configurations that give rise to different phenotype, given the genotype to phenotype map in b. The orange colour indicates ‘1’, and grey indicates ‘0’ for each genotype or phenotype element. Note that under this particular genotype to phenotype map, the phenotype vector z cannot take on the value of (1,0) under any configuration of genotype, showing the constraint from the genotype to phenotype map in our model. (d) Overview of the full model. Genotype is v, phenotype is z. The first part is a genotype to phenotype map, and the second part is a phenotype to fitness map. Selection is on phenotype, evaluated by the environment b.

Our model represents a population of hypothetical multicellular organisms evolving under Wright–Fisher dynamics. Each organism is represented with a genotype containing selector genes v acting on an effector gene regulatory network T to produce a collection of terminally differentiated cell types z. This representation constitutes an explicit genotype to phenotype map (Figure 1a). At each generation, the selector genotype mutates and mutations are evaluated by the genotype to phenotype map, resulting in a possible change in the phenotype. The phenotype and environment then determine the fitness of each individual, which the sampling of the genotype in the next generation is based on. Although the structure of the genotype–phenotype map is kept constant during simulations, the sensitivity of phenotype to genotype–robustness–can be controlled by specific parameters, either directly or by adding a robustness control locus to the genotype. Below we describe each component of the model in detail.

### 2.1 | Genotype to phenotype map

We consider a haploid organism with complete linkage of selector genes. These are the total set of selector genes in the genome which control effector genes and through them the organismal phenotype. The collection of all these selector genes is the genotype, represented by a Boolean vector v, with length L. We envision each selector gene as having two alleles, which we represent as ON $\left(v_{i}=1\right)$ or OFF $\left(v_{i}=0\right)$. Because we are interested in the pleiotropic effects of genotype on selectable traits, we model this process explicitly. We represent an individual’s multidimensional phenotype z by a Boolean vector with a collection of K traits, each of which can be identified with a cell type. Each trait considered in the model is discrete and has only two states, ON $\left(z_{i}=1\right)$ and OFF $\left(z_{i}=0\right)$, representing the presence or absence of a particular cell type that generates a selectable trait. The cell type in question might correspond to a morphological feature such as a bristle, or it might be related to the expression of an enzyme required to make pigment, giving rise to a yellow or blue body colour (Figure 1b). An effector gene which implements the effects of selector gene $v_{j}$ on cell type (trait) $z_{i}$ is denoted $t_{ij}$. We picture each effector gene as having three allelic states which promote, inhibit or have no effect on cell type $z_{i}$.

The genotype to phenotype map is represented by a one-layer neural net. Equations of this type were introduced by McCulloch and Pitts (1943), who called them ‘perceptrons’, and later applied to genetic networks by Leon Glass and collaborators (Glass & Kauffman, 1972, 1973). This perceptron maps genotype v to phenotype z using the equation

(1) $$z_{i}=\sigma \left(\gamma \sum_{j=1}^{L}t_{ij}v_{j}-h_{i}\right),$$

where $z_{i},\;v_{j}\in \{0,\;1\}$. Each component $t_{ij}\in \{-1,0,1\}$ of T represents a particular allele of an effector gene which mediates the impact of selector gene $v_{j}$ on cell type $z_{i}.\;t_{ij}=0$ means that gene $v_{j}$ is not involved in regulating cell type $z_{i}.$ $t_{ij}=1$ represents activation and $t_{ij}=-1$ represents repression. γ determines the strength of each activation or repression. Note that this contribution only occurs when the gene $v_{j}$ is ON. $h_{i}$, a real number, is the threshold for cell type $z_{i}$ to determine its phenotypic state. σ(y) is a step function such that σ(y)=0 iff y≤0, and 1 otherwise. It is evident that in order for cell type $z_{i}$ to be ON, the sum of the activation and repression strengths of all the genes $\sum_{j}\;t_{ij}v_{j}$ on cell type $z_{i}$ must be greater than the threshold value $h_{i}$.

We chose this mathematical picture in order to give the simplest possible representation of pleiotropic control of phenotype by genotype in the presence of thresholds. In order to represent the evolutionary dynamics of multicellular organisms in a tractable manner, we allow the genes in v to evolve, but not the genes that determine T. Allowing v but not T to evolve allows us to focus on the fundamental evolutionary constraints inherent to multicellularity.

Key conceptual points about our model are illustrated in Figure 1. Figure 1b shows an example of a hypothetical organism in which six genes (L=6) regulate two cell types and hence two traits (K=2). $v_{1},\;v_{4},\;v_{5},\;v_{6}$ are ON and $v_{2},\;v_{3}$ are OFF, γ is set to 1 and the actions of the genes on cell types are shown. The interactions and thresholds that determine different cell types are independently initialized. Once initialized, the genotype to phenotype map is kept constant unless the robustness parameter changes during evolution (Section III). Given the current regulation, the total activation for $z_{1}$ is below the threshold $h_{1}$, rendering $z_{1}$ OFF. The activation for (a) $z_{2}$ exceeds the threshold $h_{2}$, and therefore $z_{2}$ is ON. $z_{1}$ determines the colour of the organism, perhaps encoding an enzyme, in which the OFF state leads to the yellow colour. $z_{2}$ determines the presence or absence of a cell type containing a bristle, and as a result, its ON state gives rise to a bristle. In evolutionary simulations, only mutations in the genotype v are allowed. However, given the current genotype to phenotype map, no matter how genotype changes, only three out of four possible phenotypes can be achieved (Figure 1c). In this example, in order for $z_{1}$ to be ON, at least two genes out of $v_{1},\;v_{3}$ and $v_{4}$ have to be ON. The genotype-phenotype map thus constrains $z_{2}$ to be ON if $z_{1}$ is ON. Hence, there is no case in which $z_{1}$ is ON but $z_{2}$ is OFF. This illustrates the fact that accessible phenotypic space can be smaller than the whole phenotypic space because of developmental constraints.

### 2.2 | Phenotype to fitness map

The absolute fitness of an individual depends on both the phenotype z and the environment b, with the K components $b_{i}\in \{0,\;1\}$. The dependence of fitness on z and b could be quite complex, but in the present application, we consider the environment to specify a trivially explicit optimum phenotype, which denotes the most fit state for each cell type trait in such an environment (Figure 1d). The absolute fitness W of an individual is defined as the proportion of the K traits in a phenotype z that match those of an optimal phenotype b, so that

(2) $$W=1-\frac{D(b,\;z)}{K}$$

where D(b,z) is the Hamming distance between b and z. At the end of each generation, each individual’s relative fitness is calculated by normalizing its absolute fitness to the sum of the absolute fitnesses of all individuals in the population. The summed relative fitness of all individuals with a given genotype is the probability with which members of the next generation with that genotype are created. The list of variables is shown in Table 1.

### 2.3 | Initial conditions

In evolution simulations, described below, the initial population is uniform with a random genotype generated as follows. We randomly assign a proportion a of genes in v to be 1. The probability that each $t_{ij}$ has a non-zero value is given by the parameter c, such that $P\left(t_{ij}=1\right)=P\left(t_{ij}=-1\right)=c/2$, and $P\left(t_{ij}=0\right)=1-c$. These choices mean that the random variable $t_{ij}v_{j}$ is i.i.d. with a mean of zero and variance ac, and hence, the Central Limit Theorem indicates that for the large values of L, we consider (Table 2), $\sum_{j}\;t_{ij}v_{j}\sim 𝒩(0,acL)$. We randomly draw $h_{i}$ from the same normal distribution, so that the argument of σ in Equation (1), given by

(3) $$y_{i}=\gamma \sum_{j=1}^{L}t_{ij}v_{j}-h_{i},$$

is distributed $\sim 𝒩\left(0,\left(1+\gamma^{2}\right)acL\right)$. These values determine the initial values of z.b is set in one of three ways. For $E_{opt}$, the ‘optimal environment’, the environment b is set to be the initial phenotype z in the population. A “new environment” $E_{\text{new}}$ may be represented in two possible ways. In the first, the new environment $b^{*}$ is set to a random Boolean vector with each component having a 0.5 probability of being one. We define a second type of new environment, $b^{**}$, in which optimal fitness is guaranteed to be reachable within the genotypic space. $b^{**}$ is initialized by drawing a random genotype vector and selecting a random genotype–phenotype map, setting $b^{**}$ to be optimal for this map as was done with $E_{opt}$, but using a re-randomized genotype vector to start the simulation environment.

### 2.4 | Calculation of robustness

R, the robustness, denotes the probability that a cell type will not change as the result of a single mutation, specifically the probability that a cell type $z_{i}$ is not changed as a consequence of this mutation in $v_{j}.\;R$ has the same meaning as the parameter q used by Draghi et al. (2010), where q is an explicit adjustable parameter of the model used by these authors. In our model, by contrast, R is an emergent property of the underlying structure of the genotype to phenotype map, a model component which is absent in the cited work. Here, we instead control R indirectly by altering the argument of σ in Equation (1) by rescaling the sum $\sum_{j=1}^{L}\;t_{ij}v_{j}$ with or without a compensatory rescaling of the threshold $h_{i}$. These methods have different side effects and access different regions of the possible range of values of R, as we describe below.

The first method of controlling R makes use of the parameter γ which occurs in the argument of σ(y) in Equation (1). It is evident that the probability of altering a component of z when a component of v mutates is a monotonically increasing function of γ. In Equation (3), $y_{i}\sim 𝒩\left(0,\left(1+\gamma^{2}\right)acL\right)$, and the probability that $t_{ij}=+1$ is c/2. Hence, when $v_{k}$ mutates the probability of changing a cell type $z_{i}$ is given by

(4) $$\begin{array}{c} P=2\cdot P\left(y_{i}\right.\;\text{in}\;(-\gamma ,0)\;\text{before}\;\text{mutation})\;\cdot P(\text{when}\;v_{k}\;\text{mutates},\;y_{i}\;\text{increases}\;\text{by}\;\gamma )\\ \;=2\cdot \left[0.5-\Phi \left(-\gamma /\sqrt{\left(1+\gamma^{2}\right)acL}\right)\right]\cdot c/2\\ \;=c\left[0.5-\Phi \left(-\gamma /\sqrt{\left(1+\gamma^{2}\right)acL}\right)\right],\; \end{array}$$

where Φ(x) represents the CDF of the standard normal distribution. Then, the robustness is given by

(5) $$R=1-p=1-c\left[0.5-\Phi \left(-\gamma /\sqrt{\left(1\;+\;\gamma^{2}\right)acL}\right)\right].$$

**FIGURE 2:**
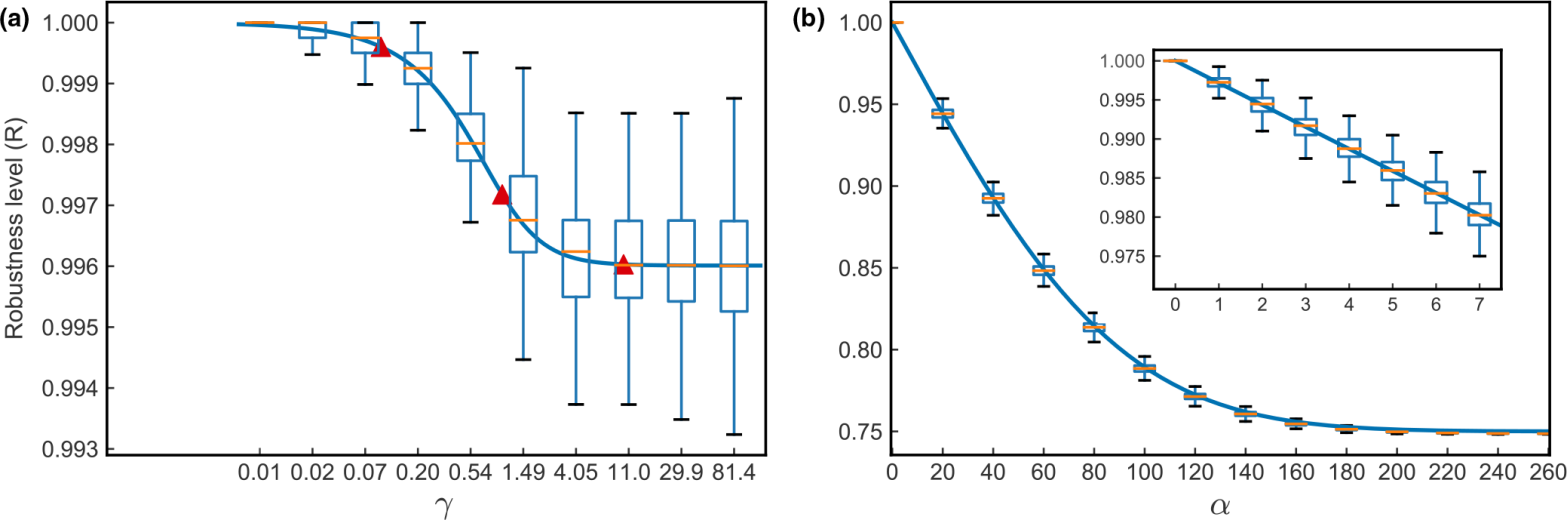
Control of robustness by γ and α. (a) Relationship of the parameter γ to robustness. Boxplots are calculations from simulations and the solid line shows the analytic result from Equation (5) with parameters c=0.5,a=0.5,L=10000,K=1000. For the simulations, we first simulate traits based on parameters. Then for each simulated trait, every bit in the genotype v is flipped and the new phenotype is calculated, the number of bits in the new phenotype compared to the initial one that are the same were recorded. A total of 1000 simulations are done for each parameter set. Red triangles mark three γ values, 0.1, 1 and 10, which are used for evolutionary simulations. (b) Relationship of the parameter α with robustness. Boxplots are calculations from simulations. The solid line shows the analytic result from Equation (7) with parameters c=0.5,a=0.5,L=10000,K=1000. Simulations are done as in. The inset shows the relationship of α to robustness for α≤7.

Because $\gamma /\sqrt{\left(1+\gamma^{2}\right)acL}$ ranges from 0 to $(acL{)}^{-1/2}$ as γ increases from 0 to ∞, it is evident that increasing γ from 0 to ∞ will cause R to decrease from 1 to values just below 1. Given the parameters in Table 2, the lowest robustness level reachable by γ is 0.996, meaning that for 1000 cell types, one mutation at most changes four cell types on average. We consider three values of γ-0.1,1 and 10–for subsequent use in simulations (Figure 2a). Note that changing γ during a simulation will change not only robustness, but also possibly the current phenotype as well, a point evident by inspection of Equation (3). In order to investigate the evolution of robustness itself, we would like a parameter that only changes the probability of mutations on future phenotypes without changing the current phenotype. In addition, this parameter allows the exploration of a wider range of values of R.

To address this problem, we set γ=1 and introduce a new parameter α in Equation (3), so that

(6) $$y_{i}=\alpha \sum_{j=1}^{L}t_{ij}v_{j}-h_{i}^{\prime },\;$$

where $h_{i}^{\prime }=(\alpha -1)\sum_{j}\;t_{ij}v_{j}+h_{i}$, thus keeping $y_{i}$ and the current phenotype z the same, but altering the probability that z will change because of a mutation in v. Then, in the initial population we have, using the same reasoning used in Equation (4),

(7) $$R=1-p=1-c[0.5-\Phi (-\alpha /\sqrt{2acL})].$$

Figure 2b shows how robustness changes by varying α. It is evident by inspection of Equation (7) and Figure 2b that as α→∞,R→0.75. As will become apparent in the Results, this range of R encompasses the biologically relevant range.

### 2.5 | Evolution simulations

The evolutionary simulation was implemented under the C++ template framework of the fwdpp model (Thornton, 2014) with modifications described below. The Eigen package (Guennebaud et al., 2010) was used to calculate genotype to phenotype map. Code is available on github (https://github.com/pyjiang/ct_fwdpp).

We evaluated evolution at different robustness levels by carrying out haploid Wright–Fisher forward simulations using the parameter values given in Table 2. In each generation, μLN mutations are introduced into the population. Each mutation corresponds to flipping a randomly selected bit in v. The phenotype is calculated for each new genotype, the fitness is calculated from Equation (2), and individuals are generated for the succeeding generation with probability proportional to relative fitness. We monitor population mean fitness as the population adapts from its initial phenotypic state under one of the three environments described above under ‘Initial Conditions’. For fixed robustness levels, 8 × 10^6^ generations were simulated under a given environment. When robustness was allowed to evolve, 1.6 × 10^7^ generations were simulated under a given environment.

We set the total number of genes L to be 10 000, the same order of magnitude as the number of genes in a typical eukaryote. For simulations with a fixed level of robustness, genes in genotype v are fully linked, with mutation rate μ per gene per generation. μ is set to be 10^−6^. The individual probability of acquiring a new mutation each generation is 0.01; with the population size (N=10000), 100 new mutations are introduced on average each generation into the population. Unless stated otherwise, the parameter values in Table 2 were used for all simulations.

For simulations with evolving levels of robustness, we add a robustness locus r which mutates at rate $\mu_{r}$. Eight levels of robustness are possible, represented by three bits (Table S2). Following mutation, the robustness locus is evaluated first to determine the appropriate α value for that individual, which is then applied to the genotype v to calculate the phenotype. The genes in v remain completely linked, but recombination is possible between the genotype v and the robustness locus r. The recombination rate r can range from 0 to 0.5. Recombination was implemented by exchanging robustness loci in Poisson (2rN) haploid pairs.

## 3 | RESULTS

As shown in the description of the model, the value of robustness R under the genotype to phenotype map can be controlled in three ways, which we consider in the three sections below. In Section I, we first explore evolution dynamics with three levels of robustness within a highly restricted robustness space regulated by γ. In Section II, we explore the relationship of evolution with different levels of robustness under a larger robustness space regulated by α. Lastly, in Section III, we encode robustness with a genetic locus and let it co-evolve with the phenotype under selection.

### 3.1. | Section I: Evolution near maximal robustness

In this section, we begin our analysis by considering very small departures from the limiting case of maximum robustness R=1. Although all levels of robustness considered in this section are high in absolute value, they have significantly different effects on evolution. In the presentation below, we denote these regimes relative to one another as high, medium and low robustness. These terms describe values of R=0.9996,0.9972 and 0.9960, respectively, obtained by setting γ to 0.1, 1 and 10.

#### 3.1.1 | Evolution in a constant, optimal environment $\left(E_{opt}\right)$

We first explore which level of robustness would be evolutionarily preferable when its current phenotype has already adapted to such an environment, denoted by $E_{opt}$.

**FIGURE 3:**
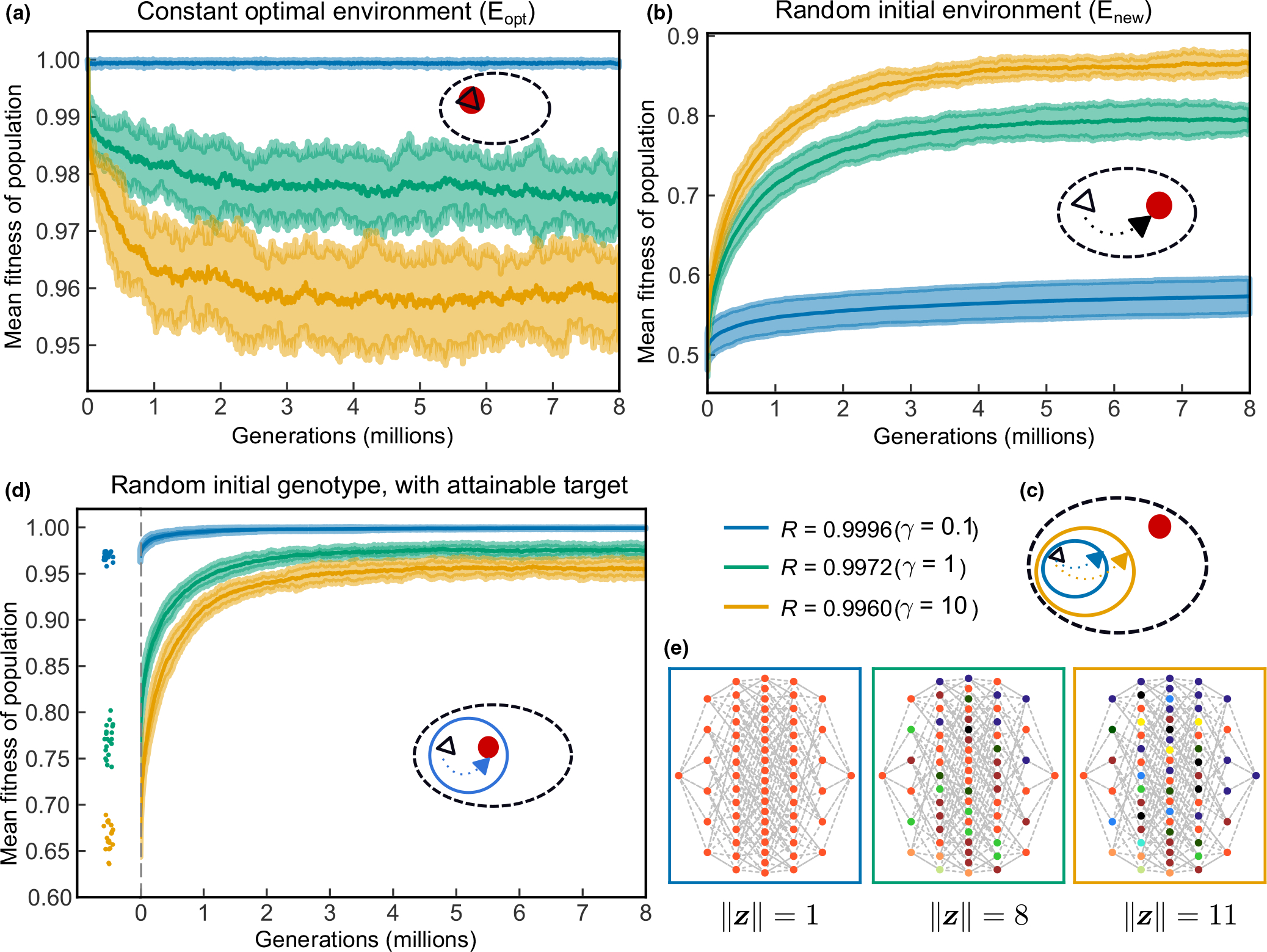
Different evolutionary trajectories with a fixed level of robustness by γ. Schematic plots (oval-shaped graphs) in a-d indicate what kind of evolutionary regimes in the phenotypic space the simulations are in. Black dashed oval indicates the whole phenotypic space. Black triangle represents the initial phenotype of the starting population. Red solid circle indicates the optimal phenotype specified for each environment. Circle with either blue or yellow colour represents the total phenotypic space for R=0.9996 (γ=0.1) and R=0.9960 (γ=10) respectively. (a) Mean fitness changes over time for populations with different robustness levels under $E_{opt}$. A total of 20 simulations were done for each level of robustness. The thick solid lines show the mean of the simulations at each generation. The upper and lower boundaries are 1.5 standard deviation on each side of the mean (same in b, d). Results from every 5000th generation are plotted due to the large number of generation simulated (same in b, d). (b) The change in mean fitness over time for initially homogeneous populations with differing robustness under a random new environment $E_{\text{new.}}$. (c) Schematic plot illustrates high robustness population have a smaller phenotypic space compared to low robustness ones. (d) Mean fitness changes over time for populations initialized with a ‘attainable’ random environment, where this new environment $b^{**}$ is initialized using a random genotype, with the same genotype to phenotype map as the initialized population. To the left of the vertical dashed line shows the initial mean fitness of each population in the new attainable random environment. Each dot represents one simulation, with jittering. (e) One simulation showing phenotypic space for all genotype combinations for the three robustness levels for K=6,L=6. Each circle represents a unique genotype, and each column indicates different total number of ‘1’s in the genotype vector (from left to right, zero ‘1’s to six ‘1’s). Each genotype is connected to its neighbouring genotype that has one bit of difference in dashed lines. One set of randomly generated T and h was used for all γ values and for all combinations of genotype. For different γ values, the T was scaled to γT, according to the Equation (1). The corresponding phenotype for each genotype was calculated. Each Boolean phenotype vector was then converted to an integer value, from which each unique value is assigned to a colour. The numbers at the bottom indicates the unique number of different phenotypes in this simulation, that is, size of the phenotypic space under a particular robustness level.

Figure 3a shows mean population fitness changes across with high, medium and low robustness (in blue, green and yellow). Populations are initially fixed for the optimal genotype and are allowed to evolve with a fixed level of robustness until a plateau in population mean fitness is observed. With high robustness (R=0.9996), the population mean fitness remains close to the optimal value ($\overset{‾}{W}=0.99929,\;\sigma =0.00025$), while when robustness is low R=0.9960, the population mean fitness equilibrates at a lower value ($\overset{‾}{w}=0.95869,\;\sigma =0.00478$). Under $E_{opt}$, high robustness is selectively favoured over low robustness. Since the optimal phenotype is fixed in the initial population, mutations are deleterious initially (Figure S1C), and even at equilibrium, the distribution of selective effects remains negatively skewed with a negative mean (Figure S1D). The reduction in population mean fitness is driven mostly by fixed mutations rather than segregating mutations (Figure S2). In fact, a greater proportion of mutations are purged from the population when robustness is low, resulting in lower mean population heterozygosity (Figure S1A). The loss of fitness when robustness is lower, we hypothesize, may be a consequence of genome-wide hitchhiking effects of selection against deleterious mutation under our model of complete linkage of loci (Charlesworth, 2013; Charlesworth et al., 1993; Haigh, 1978).

We also investigated evolution under $E_{opt}$ for all combinations of values of K and L∈{100,1000,10000}, with all other parameters being the same (Figure S3. The corresponding robustness levels R can be found in Table S1.) Within the robustness range determined by γ, there is an overall decline in population fitness with decreasing robustness, and the sensitivity of population fitness to robustness is greater with larger number of genes. One combination of parameter values (K=10000,L=1000), however, produces an anomalous result in that this pattern reverses, with only high robustness having reduced fitness (γ=0.1; Figure S3F). This behaviour may be a manifestation of Muller’s Ratchet wherein, after the population exceeds a threshold number of deleterious mutations, initially hidden by robustness, mean fitness declines precipitously.

#### 3.1.2 | Evolution following a sudden environmental shift $\left(E_{\text{new}}\right)$

What will the relationship between robustness and evolution be when there is a sudden environmental shift? We first explore this question by randomly creating a new environment $b^{*}$, and challenge the homogeneously initialized population to evolve towards this optimum regardless of its current phenotype. The expected initial mean fitness of population is 0.5 according to our fitness definition. We find that populations with low robustness adapt more quickly than those with high robustness (Figure 3b). Low robustness populations also reach substantially higher equilibrium mean fitness than high robustness populations. Both of these observations–the initial rate and the final mean fitness of population–have monotonic relationships with robustness (Figure S4). The increase of population mean fitness is driven mostly by fixed mutations (Figure S2).

The aforementioned simulations were initialized with a genetically homogeneous population, whereas most populations will be genetically variable prior to an environmental shift. We therefore investigated whether initial genetic variability affects the ability of a population to evolve to a new optimum phenotype, noting that robust populations are more genetically variable than less robust populations (Figure S1A). We subject populations to evolution under $E_{opt}$ for 8 × 10^6^ generations (Figure 3a) before applying a change in environment (a randomly chosen $b^{*},\;E_{\text{new}}$) at the very next generation. We then allowed the populations to adapt to this new environment for another 8 × 10^6^ generations. The population evolutionary dynamics after the shift (Figure S1B) show little difference compared to populations starting with a single genotype (Figure 3b vs. Figure S1B). Thus, there is no initial advantage under this framework in highly robust populations to have higher genetic diversity in terms of their ability to adapt to a novel environment. This is because the genotype to phenotype map is kept constant while changing the environment. Although high robustness populations possess greater initial genetic diversity at the onset of environmental change, high robustness is restricting their phenotypic variation and therefore hinder selection that can act when adapting to a new environment.

The distribution of selection coefficients, s, when adapting towards a new optimum, is initially close to symmetric under our model for high and medium robustness, with a positive mean for low robustness (Figure S1E). Moreover, low robustness populations have a larger variance in fitness, which accounts for their more rapid initial increase in fitness as predicted by Fisher’s fundamental theorem of natural selection.

High robustness populations also reach a lower fitness plateau than low robustness populations. This is surprising because classic population genetic models predict a mutation-selection balance such that no matter whether populations start high or low in their initial fitness, at equilibrium, they would reach the same level of population mean fitness (Goyal et al., 2012). However, this does not hold in our model. We hypothesized that high robustness results in a smaller phenotypic space than low robustness, and that when a random phenotype target is drawn from the universe of possible phenotypes, it will typically be outside of the realizable phenotypic range of the population. Since low robustness explores a larger phenotypic space, it can evolve to a phenotype closer to the random phenotypic target (Figure 3c). We create a schematic notation to illustrate this idea, and add it to each of the simulation plots. The oval with black dashed lines represents the whole phenotypic space. The circle with a solid line indicates the phenotypic space with a certain level of robustness. The black triangle represents the phenotypic state of the current population. The red solid circle represents the environment’s most preferred phenotypic state.

To test this hypothesis, we set up a different initial ‘random’ optimal environment $b^{**}$ such that its corresponding optimal phenotype is reachable within each population’s genotypic search space (Figure 3d). The schematic in the inset of Figure 3d shows that the $b^{**}$ is within the phenotypic space of high robustness as an example. Under this scheme, populations achieved similar equilibrium mean fitness for each robustness level as when they started with the optimal phenotype (Figure 3a,d). This indicates that when the phenotypic target is achievable within the starting population’s phenotypic space, the population is able to evolve to the new optimum and reach a mutation-selection balance. Notably, when examining the initial fitness of populations under $b^{**}$, high robustness populations have a phenotype more similar to their parental genotypes, resulting a higher initial fitness values (Figure 3d, initial fitness). All these results support our hypothesis.

Since it is not practical to enumerate the complete phenotypic space for L=10000 genotypes, we enumerate all combinations of genotype and phenotype under the three robustness levels with lower dimensions at L=6,K=6. Phenotypes are generated from all possible genotypes with the same genotype to phenotype map, except the robustness parameter. Figure 3e shows one example simulation. It is clear that high robustness produces a much smaller phenotypic space than low robustness, supporting our hypothesis.

### 3.2 | Section II: Evolution under a wider range of robustness

#### 3.2.1 | Evolution under $E_{opt}$ and $E_{\text{new}}$

α allows us to explore a wider range of robustness R, from just below 1 to 0.75 (Figure 2b). In this section, we investigate robustness regulated by α for populations under $E_{opt}$ and $E_{\text{new}}$.

**FIGURE 4:**
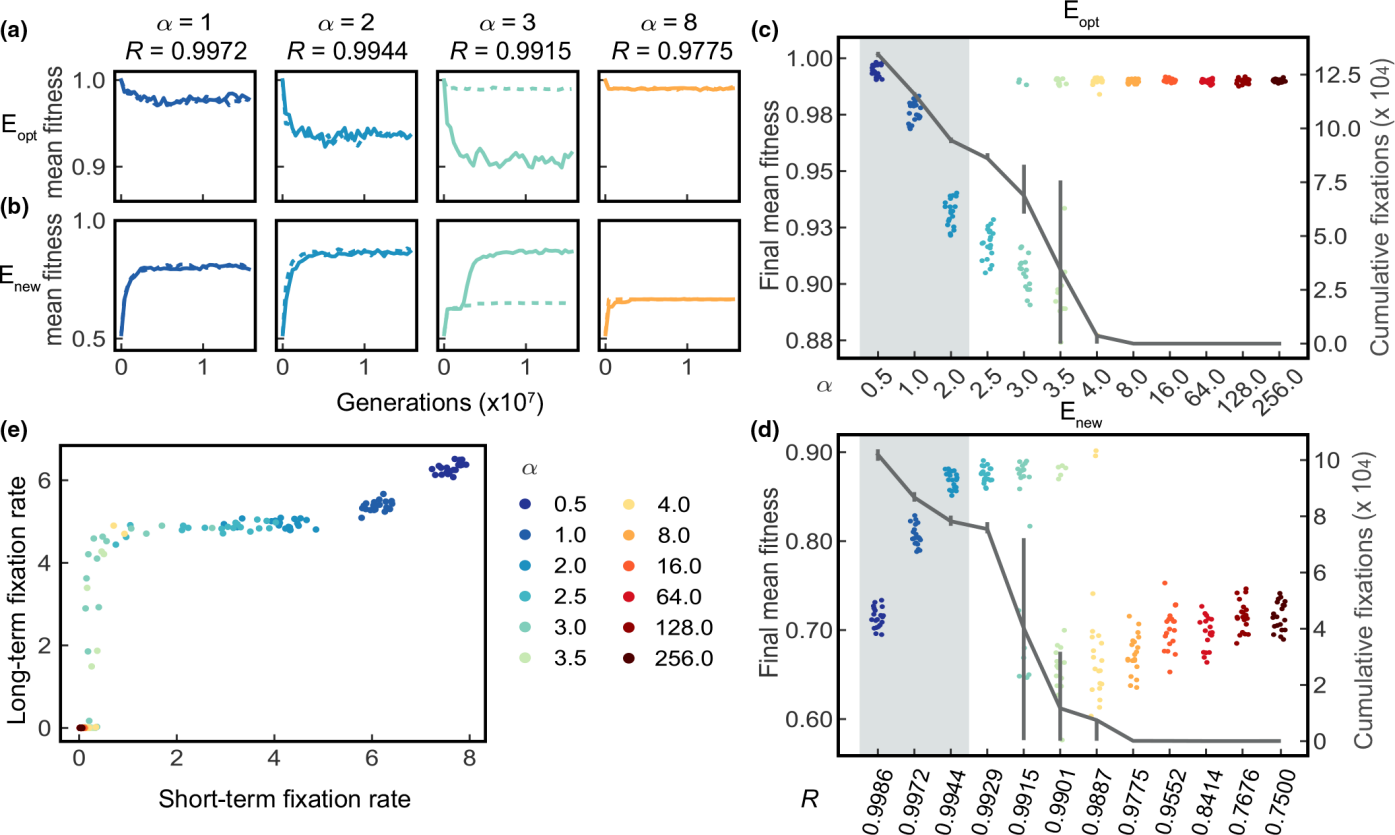
Populations showing non-linear relationship with larger range of robustness regulated by α under constant optimal or random initial environment. (a) Two representative simulations showing mean fitness of population changes over time for α values 1,2,3 and 8 under $E_{\text{opt}}$ One simulation is shown by a solid line and the other is by a dashed line, over $1.6\times 10^{7}$ generations. (Results from every 400000th generation are plotted due to large number of generation simulated. Same in b.) (b) Two representative simulations showing mean fitness of population changes over time for α values of 1,2,3 and 8 under $E_{\text{new.}}$. The x axis of c and d are the same, with c denoting the α values, and d denoting the corresponding the robustness levels R. (c) Behaviours of final population mean fitness at $1.6\times 10^{7}$ generations with different α under $E_{opt}$ for 20 simulations at each α value. Different colours represent different α values. Note this colour scheme for different α values are used consistently in Figures 5 and 6. Jittering is used to display points with the same α values. The line shows the mean number of fixations at the end of the simulations with each α value. The error bars show 15% and 85% of the final fixation numbers. The light grey rectangle-shaded area shows the range of robustness considered in Section I. (d) Same as c under $E_{\text{new}}$. (e) Short-term fixation rate versus long-term fixation rate for populations with different α. Fixation rate is calculated as the average number of fixations per 1000 generations given a period of time. Short-term fixation rate is the average fixation rate for the initial 10^6^ generations, and the long-term fixation rate is the average fixation rate from generation 1 000 001 to generation 16 000 000.

Rather than the monotonic relationship of robustness to fitness found when varying γ from Section I (Figure 3a,b), we observe more complicated dynamics with a bifurcation in final mean fitness at intermediate α, and an inversion in the relationship between robustness and evolution when robustness decreases further. Typical trajectories are summarized in Figure 4a,b, with two representative simulation trajectories for four robustness values R=0.9972,0.9944,0.9915,0.9775 specified by α=1,2,3 and 8, respectively, under $E_{\text{opt}}$ or $E_{\text{new}}$ (Figures S5 and S6 show trajectories for all α values). For simplicity, we will describe results by α values. When α varies between 1 and 2, the evolution dynamics is similar to that in Section I. This happens because the parameter space of robustness R explored by α within this range is similar to that of γ. However, when α=3, we saw a novel bifurcating behaviour of evolution trajectories. Under $E_{opt}$, populations either remain close to the optimal fitness value (Figure 4a, dotted line), or drop to a lower fitness value (solid line). When challenged to adapt to $E_{\text{new}}$, populations either reach a higher fitness value (Figure 4b, solid line) or are trapped with suboptimal phenotypes (dotted line). When robustness decreases even further, interestingly, populations remain close to the optimal fitness in $E_{\text{opt}}$ when α=8. This reverses the relationship of evolution with robustness. Similarly, such a reverse in the relationship is reflected in $E_{\text{new}}$, populations with lower robustness are unable to reach high fitness when challenged with $E_{\text{new.}}$.

Mean fitness at the end of each simulation is shown in Figure 4c, d for all α value tested. The grey shaded area shows the corresponding robustness in Section I. For $E_{opt}$, further decreasing robustness R by increasing α beyond 8 does not change its behaviour. For $E_{new}$, increasing α beyond 8 only slightly increases the final mean fitness, but mean fitness stabilizes for α≥128. Specifically, we find that low robustness (α≥8) populations, though they eventually adapt poorly to a novel environment, initially adapt more quickly than high robustness populations (Figure 4d and Figure S8).

We sought to find the cause of the bifurcation at intermediate α and the inverted behaviour when robustness decreases further. We suspect it could be attributable to a different number of fixations under different robustness levels, so we examined the total number of fixations for each α value at the end of the simulations (Figure 4c,d, grey lines). We find a similar pattern for the cumulative number of fixations for $E_{\text{opt}}$ and $E_{\text{new}}$ simulations–the total number of fixations decreases with decreasing robustness, and becomes almost zero when α≥8. We also observed an increased variance in the number of fixations for α values between 3 and 4, which correlates with the bifurcation behaviour in the final mean fitness of each population. We also compared the cumulative number of fixations against final population mean fitness under both $E_{opt}$ and $E_{\text{new}}$ for α≥2.5 (Figure S9). We observe two non-overlapping clusters of population final mean fitness, one high and one low, separated by the number of fixations. For $E_{opt}$, populations that achieve high fitness fix no mutations (Figure S10), while the ones that fix many mutations have a lower fitness. For $E_{\text{new}}$, populations that fix few mutations remain at a low fitness while ones with many fixations achieve a higher fitness.

Note for $E_{\text{new}}$ at α=8, there are some fixations in the first 2 × 10^6^ generations but populations nevertheless remain trapped at low fitness (Figure S11). We therefore suspect fixation rate at early and late stages can affect population dynamics differently. We quantify fixation rate as the average number of fixations per 1000 generations, and calculate early and late fixation rate in the first 10^6^ generations or the next 15 × 10^6^ generations (Figure 4e). When α≤2, the fixation rate is high and differs little between early and late. When α≥8, both early and late fixation rates are close to 0, with some having low early fixation rates. In contrast, for intermediate α values (2.5≤α≤4), the fixation rate is low early and late, or it can be low early but jump to a higher value in the late period. This latter situation represents evolutionary trajectories leading to high fitness. Whether or not populations can gain a positive long-term fixation rate determines the bifurcation behaviour.

### 3.3 | Section III: A co-evolved locus that controls robustness

To gain a better insight of how robustness could evolve indirectly when only the phenotype is under selection, we introduce a mutable locus, r, controlling robustness levels by mapping its genotype to parameter α. The reason we chose to change α is because it has the desirable property of only affecting sensitivity of mutations on phenotypes while keeping the current phenotype the same. We encode r with eight levels of robustness, ranging from 0.5 to 8 (the first eight α values in Figure 4c) since the evolutionary dynamic behaviour for α larger than 8 is qualitatively similar to α=8 from Section II. Three bits are used to encode the eight states (Table S2). A mutation in the robustness locus alters a single bit, as is the case for the rest of the genotype. The mutation rate of the robustness locus is set to $\mu_{r}=10^{-5}$. The population is initialized with equal frequencies of the eight robustness alleles. The population then evolves under the same two environmental scenarios $E_{\text{opt}}$ and $E_{\text{new}}$.

**FIGURE 5:**
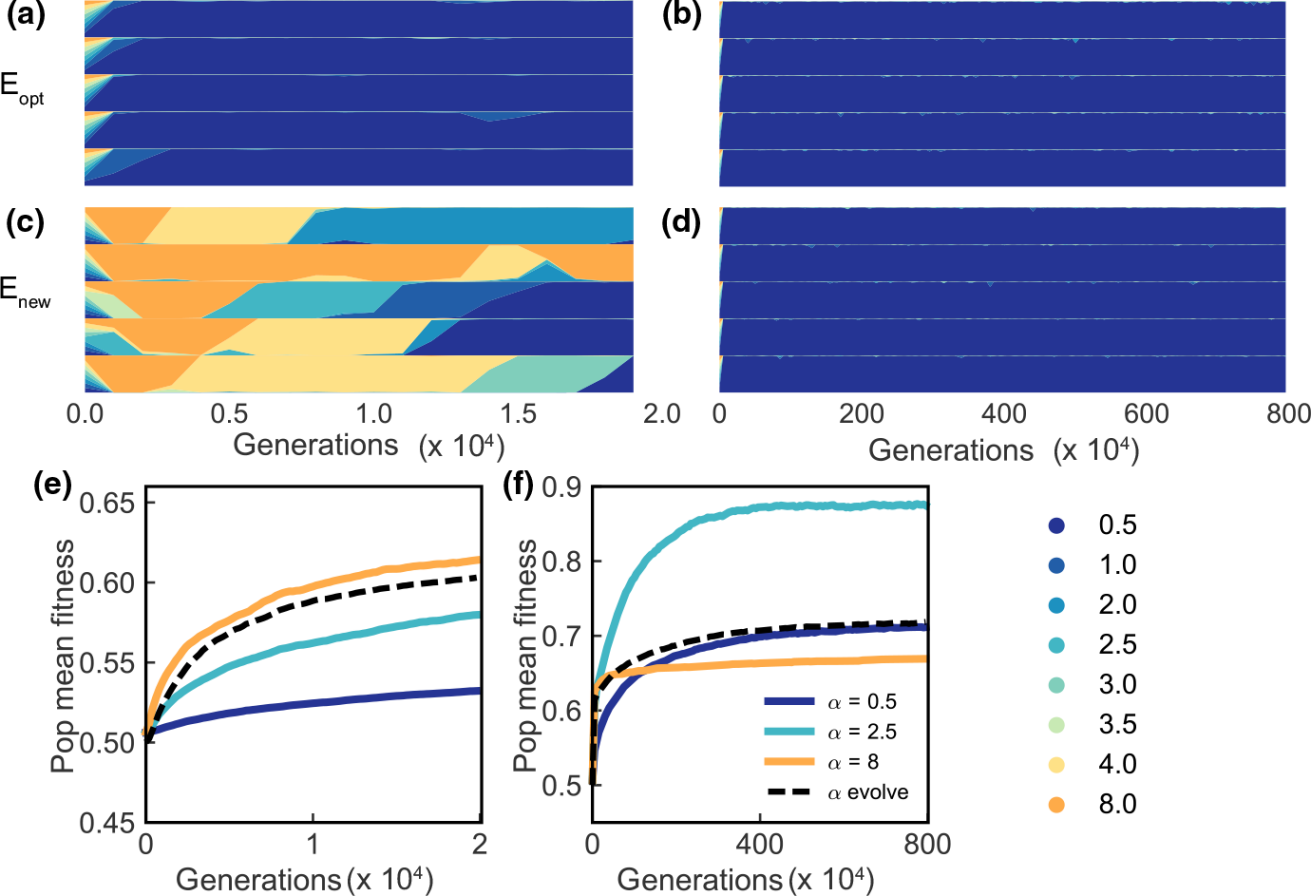
Population dynamics with evolving robustness. (a-d) The frequencies of the eight robustness genotypes in five simulations with evolving robustness. Each horizontal bar shows one simulation result. For a and c, robustness genotype frequency from every 1000th generation are plotted. For b and d, robustness genotype frequency from every 50000th generation are plotted. (a and b) The robustness genotype frequency changes over time under $E_{opt}$, with a showing the change over the first 2 × 10^4^ generations, and b for 8 × 10^6^ generations. c and d show the robustness genotype frequency changes over time under $E_{\text{new}}$, with c showing the change over the first 2 × 10^4^ generations, and d for 8 × 10^6^ generations. (e and f) Mean fitness change comparison for populations with different fixed levels of robustness and one simulation with evolving robustness under $E_{\text{new.}}$. Solid line shows the mean value of the mean fitness of 20 simulations with a fixed level of robustness, and the dashed line shows the mean fitness of one simulation with evolving robustness. (e) The comparison for the first 2 × 10^4^ generations and every 200th generation is plotted. (f) The comparison for 8 × 10^6^ generations and every 50000th generation is plotted.

Our initial simulations were carried out with the robustness locus completely linked to genotype v. Under $E_{opt}$, the most robust genotype (α=0.5) goes to fixation in every simulation we examined (n=20, Figure 5a,b show five instances). We note, however, this behaviour is not consistent with the long-term population mean fitness changes with a fixed level of robustness (Figure 4a,c), that high mean population fitness is achieved on either side of the α bifurcation, with the least robust population (α=8) actually having the highest fitness. Since the high robustness allele (α=0.5) fixes rather quickly, we suspect it could be due to short-term fitness advantages at early stages of the evolutionary simulations. We therefore examined the population dynamics with constant α in the initial 200 generations in $E_{opt}$, and indeed found initial fitness decrease monotonically with robustness (Figure S12).

For populations adapting to $E_{\text{new}}$, the lowest robustness allele (α=8) is selectively favoured initially (Figure 5c), consistent with initial population dynamics with constant robustness under this environment (Figure S8). We compare the evolution trajectories of populations with an evolved robustness locus with a fixed level of robustness (Figure 5e,f). Figure 5e shows the evolution dynamics in the first 2 × 10^4^ generations. The populations with evolving robustness behave similarly to the one with fixed α=8, suggesting low robustness is preferred at the onset of adaptation. However, as the adaptive process continues, the highest robustness genotype (α=0.5) takes over the population permanently (Figure 5f). Population mean fitness then proceeds to increase along the trajectory given for fixed α=0.5, to a stable mean population fitness of ≈0.7. Thereafter, it does not improve any further. Surprisingly, this is not nearly the highest fitness the population could have achieved. For example, with fixed α=2.5, mean fitness rises to nearly $\overset{‾}{w}=0.9$ (Figure 5f). The high robustness genotype fixed early despite the fact that it prevents the population from evolving to a higher fitness.

**FIGURE 6:**
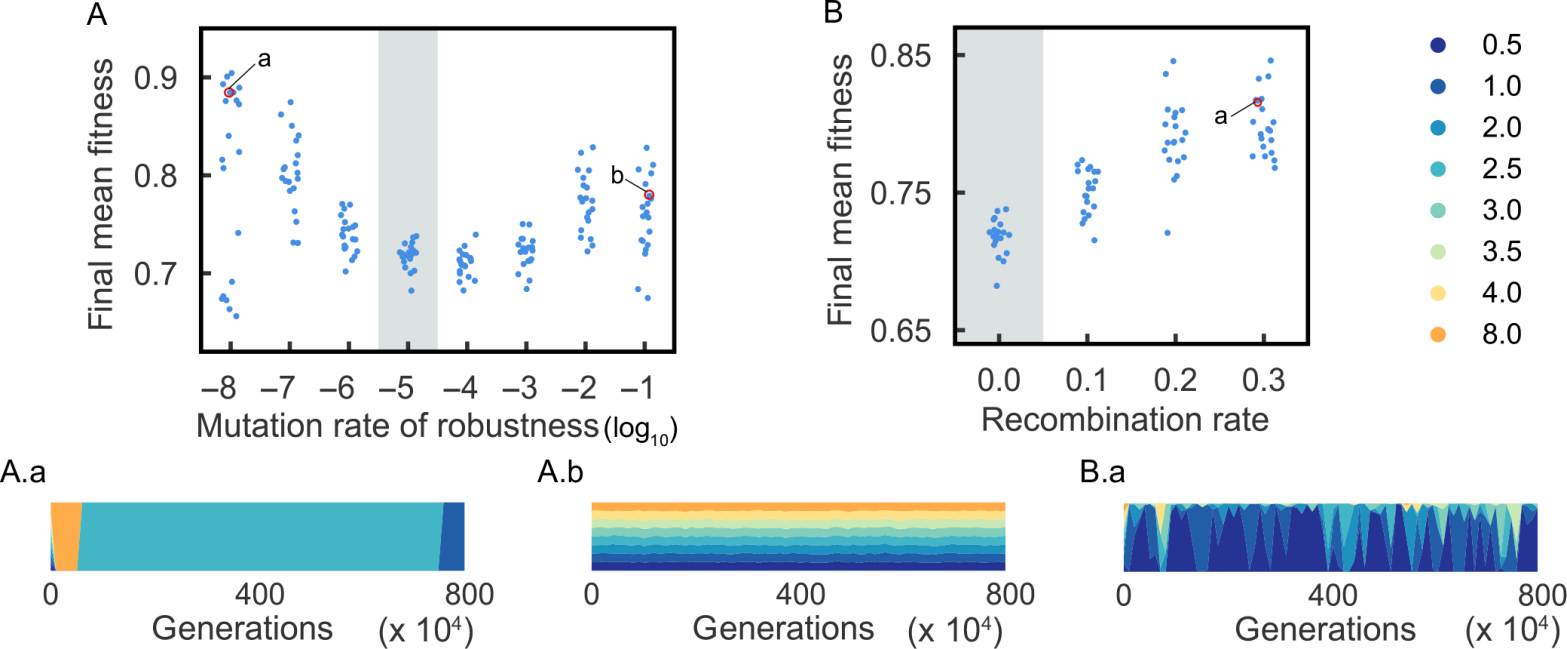
Mutation and recombination rate of robustness locus affects the adaptation process. Relationship of mutation rate of robustness locus r and recombination between robustness locus r and genotype v to evolution under $E_{\text{new.}}$. The light grey area shows the results with the parameters used in Figure 5. (A) Final mean fitness of populations after 8 × 10^6^ generations with different mutation rates of the robustness locus. Each dot represents one simulation. Jittering is applied for simulations with the same mutation rate. (A.a) One example simulation of robustness genotype frequency changes over time, with the mutation rate of robustness locus being 10^−8^. (A.b) One example simulation of robustness genotype frequency changes over time, with the mutation rate of robustness locus being 0.1. (B) Final mean fitness of populations after 8 × 10^6^ generations with different recombination rates between the robustness locus r and genotype v. The recombination rate is the probability that a recombination event occurs between a randomly chosen pair of haploids. (B.a) One example simulation of robustness genotype frequency changes over time, with recombination rate being 0.3 per individual per generation.

We hypothesized that this behaviour could depend on the specific parameterization or assumptions of the model. We investigated these possibilities by varying the mutation rate of the robustness locus r and also allowing the robustness locus to recombine with v. We found that decreasing or increasing mutation rates at the robustness locus increases the equilibrium mean fitness (Figure 6A), but they achieve this through different means. A lower mutation rate prolongs the time to elimination of low robustness alleles, allowing greater time to select for a more highly adapted phenotype (Figure 6A.a). A higher mutation rate increases the number of segregating robustness alleles (Figure 6A.b). Recombination between the robustness locus and v also increases the final mean fitness of the population (Figure 6B). Indeed, the highest mean fitness is reached when at the maximum recombination rate (r=0.3) between r and v in the simulation (Figure 6B.a). These results show that the dynamics of adaptation when robustness can also simultaneously evolve is sensitive to the genetic parameters (i.e. mutation and recombination rates) of the system.

## 4 | DISCUSSION

We believe that this study has established several new insights into the relationship between canalization and evolution in multicellular and other eukaryotic organisms. We assayed evolvability by comparing changes in the mean fitnesses of populations with differing levels of robustness following an abrupt environmental change. As established previously (Draghi et al., 2010), we have shown at a qualitative level that maximum evolvability occurs at intermediate levels of robustness. In contrast to this result, at a quantitative level, we find that in multicellular and other hierarchically structured organisms, evolvability occurs at much higher levels of robustness in than was reported previously (Draghi et al., 2010) and is much more sharply peaked. Moreover, this peak in evolvability is discontinuous, and appears to be a consequence of disequilibrium caused by variation in the fixation rate. When robustness itself can evolve, we find that it attains levels which maximize relative fitness, thus insuring lower levels of robustness immediately following an environmental change and higher in a constant environment. In this section, we present our main theoretical findings, compare them with experiment and then consider the limitations and implications of this study.

### 4.1 | Theoretical findings

At the theoretical level, the model used here has extended the familiar population genetics picture in three ways. First, we provide a mathematical representation of phenotype as the consequence of the existence of multiple cell types in a multicellular organism, allowing the treatment of varying degrees of pleiotropy and epistasis in the genotype-phenotype map. Second, we provide an explicit representation of the emergence of fitness as a result of interactions between the phenotype and the environment. Third, we provide a quantitative and controllable measure of robustness R, the probability that a mutation leaves the phenotype unchanged. Although the first two features of this model have been used elsewhere (Grigoriev et al., 2014; Reinitz et al., 2019), the ability to control the level of robustness and measure its effect of evolvability under environmental change is a novel feature of this work. The explicit representation of environment generalizes different evolutionary regimes, without making arbitrary assumptions about the selection coefficient. The use of R enables us to make precise and quantitative comparisons with other studies. This is a key feature of this work, since it is often possible to raise or lower robustness in a model or experimental system, but systematic comparisons of results are difficult. Our ability to control R in small gradations near unity with the parameter γ and over a wider range by varying α allows precise control of robustness.

Our results support the idea that high robustness provides the best fitness in constant environments. This is in accordance with previous modelling results that found that high robustness is produced as a consequence of stabilizing selection in network models applied to a single cell studied in the context of population genetics (Bullaughey, 2011; Siegal & Bergman, 2002; Wagner, 1996; Wagner et al., 1997). The maintenance of a steady average fitness level in the modelled haploid population also supports the picture that beneficial and deleterious mutations tend to an attracting steady state (Goyal et al., 2012) in which the fitness of the steady state slowly decreases as R decreases from 1.

Our central result is that in response to environmental change, the optimal level of evolvability is achieved at values of R around 0.99. This result sharply differs from that of Draghi et al. (2010). The parameter q used by these authors has the same meaning as R used here, but we retain the difference in notation since q is directly set as a parameter while we control R indirectly.

Draghi and co-workers found a broad peak of evolvability between q=0.8 and q=0.2. This difference between our findings is a result of the constraint of multicellularity. In a multicellular organism, a single mutation will change many cellular states though pleiotropy, and although these changes may not be particularly deleterious to individual cells, they can have a drastic effect on fitness at the organismal level. Draghi et al. (2010) used a model inspired by and tested against the phenotype of RNA secondary structure, inherently a much less constrained system. Thus, the difference between the levels of robustness optimal for evolvability in these two studies is, in retrospect, both unsurprising and natural both in terms of the optimal level of robustness and the narrowness of the peak obtained. We predict that as further studies are conducted with models that represent the increasing level of hierarchical complexity found in phenotypes extending from RNA and protein structures to prokaryotes to single-celled eukaryotes to multicellular organisms, greater hierarchical complexity will lead to increasingly high and narrowly constrained robustness at optimum evolvability.

The level of optimal robustness for evolvability is not only much higher than that reported by Draghi et al. (2010), but also occurs by a different mechanism. Inspection of Figure 4d reveals that upon challenge with a new environment, the resulting mean fitness increases as R decreases to =0.9929 (α=2.5), but as R decreases through values of 0.9915 (α=3), 0.9901 (α=3.5) and 0.9887 (α=4), two distinct branches are visible with sharply differing fitnesses. The upper branch increases in fitness to R=0.9887, but becomes more improbable and contains fewer and fewer points as R decreases, and below R=0.9887 no instances of high fitness are seen in our simulations.

In Draghi et al. (2010, Figure 2), the corresponding curve is single valued and smooth. Biologically, our result is the effect of contingent processes in evolution. It is well known that mutations in developmental selector genes can have major effects on phenotype (Lewis, 1978, e.g.) that may be related to macroevolutionary processes, but that occurrences of such mutations that are not severely deleterious or even lethal are very rare. At the theoretical level, our results indicate that evolvability of multicellular organisms must be treated with birth-death equations (‘master equations’) using jump Markov processes. This fact is a consequence of the nature of the modelled system. Draghi et al. (2010, S.I.) formulated their model in terms of the birth-death Moran process and showed that it was well approximated by a Fokker-Planck equation in continuous time. The conditions under which such approximations can be made (Gillespie, 1992, 2000) do not apply to our model or any model in which a rare stochastic event can send the system down one of two or more alternative pathways. We interpret the bifurcation behaviour in Figure 4d as analogous to the stochastically determined selection of developmental pathways of phage λ (Arkin et al., 1998). A possible analogy in an evolutionary context is a speciation event, although detailed exploration of this interpretation is beyond the scope of this work. We do suggest that, given the enormous number of bifurcations found in the tree of life, this inapplicability of the Fokker-Planck diffusion equation is likely to be a general feature of future studies of adaptation.

The results reported in Section III indicate that when robustness itself is under genetic control, robustness is selected to an initial level which is high for constant environments and lower after an abrupt environmental change. Thus, as shown in Figure 5a–d, under the constant environment $E_{opt}$, the highest level of robustness is selected for, but after an abrupt change to $E_{\text{new}}$, there is a transient period of low robustness, followed by a return to robustness levels optimal for a constant environment. The responsiveness of robustness to change or stasis of the environment was not phenotypically optimal, however (Figure 5e,f), because selection for the lowest level of robustness in the steady state appears to freeze in suboptimal phenotypes over the long term by premature fixation.

We believe that this premature fixation phenomenon reveals a role for recombination. Premature fixation can be alleviated by either permitting recombination between the robustness control locus and other genes or by raising or lowering the mutation rate. We view the explanation in terms of altered mutation rate as unrealistic. Lengthening the time for purifying selection requires decreasing the mutation rate by two to three orders of magnitude from 10^−5^ to an unrealistic level of 10^−7^ − 10^−8^ per generation, while improving fitness by ensuring that a diverse population of robustness alleles is maintained by mutation requires increasing the mutation rate by three or four orders of magnitude to a similarly unrealistic level of 10^−1^ − 10^−2^ per generation. We consider our finding that recombination between the robustness control locus r and v relieves premature fixation to be mechanistically reasonable, particularly if the high recombination rate needed, 0.2–0.3, is interpreted mechanistically as r and v segregating on different chromosomes, which were not explicitly represented in the model.

There is a notable similarity between the behaviour of the modifier locus described in Section III and the existing theory of modifier alleles controlling the mutation rate, given the analogy that a reduction in mutation rate and an increase in robustness both decrease fitness variations. The theory of mutation modifiers predicts that such modifiers will evolve to maximize the mean fitness (Karlin & McGregor, 1974; Liberman & Feldman, 1986). The Unified Reduction Principle (Altenberg et al., 2017), a generalization of these ideas, predicts that in equilibrium, the allele with the lowest mutation rate will take over, regardless of the type of selection. We saw a similar equilibrium behaviour in our model in that the modifier allele with the highest robustness takes over the population in both of the selection regimes we considered. As is the case with classic modifier theory, our model assumes the modifier locus controlling robustness is not directly under selection. However, there is a major difference between our model and the population genetics modifier theory, which has a single equilibrium mean fitness of the population (Goyal et al., 2012). Our model, on the other hand, will result in the different equilibrium mean fitness with different levels of robustness because of constraints arising from the genotype-to-phenotype map (Figures 3 and 4). The possible unification of classical modifier theory with the results presented here into a more generalized theory is an important open problem for future study.

### 4.2 | Applicability to hierarchical phenotypes in eukaryotes

Although our study was motivated by consideration of the constraints inherent to the evolution of multicellular organisms, the coarse-grained model used is susceptible to broader interpretation. Our central result applies to any situation in which multiple genes control multiple traits in a many-to-many manner. Such levels of pleiotropy are characteristic of hierarchical organization, and in such cases, a single mutation will affect a variety of traits, some adaptively and some deleteriously. Large deleterious changes–possibly leading to lethality–in some traits controlled by a given gene will nullify large adaptive changes in others. In such a situation, adaptation requires that phenotype be strongly buffered against mutation. High pleiotropy under hierarchical organization does not apply in relatively nonhierarchical systems. Nonpleiotropic counterexamples include the trait of tRNA secondary structure which is under the control of a single gene, as well the *E. coli* trait of ability to use lactose as a sole carbon source, under the control of the permease gene lacY and β-galactosidase gene *lacZ*. We would expect that this requirement for buffered effects of mutations to increase along a gradient of depth of hierarchical organization from prokaryotes to eukaryotes to multicellular organisms. Unicellular eukaryotes such as yeast are much more amenable to experimental study of evolution than multicellular organisms, affording an opportunity for experimental corroboration.

### 4.3 | Experimental corroboration in eukaryotes

We have discussed our major findings point by point in terms of theory because the amount of evolutionary change directly observable in multicellular organisms is limited by the time scale of feasible experiments. These limitations do not exist in unicellular eukaryotes like yeast or tissue culture lines. Although quantitative measurements of mutational robustness cannot as yet be performed in experimental systems, robustness can be increased or decreased in an experimental context. Our findings about the genetic control of robustness adduced above also apply in experimental systems, which we now consider.

In agreement with the results reported here, experimental studies of natural populations at the morphological and molecular levels exhibit evidence of high robustness when average population fitness is near its optimum. A study of morphological phenotype in yeast indicated that Hsp90 not only buffers standing genetic variation in populations under selection, but also displays the opposite effect for de novo mutations from lines where mutations have not been subject to stabilizing selection (Geiler-Samerotte et al., 2016). This can be interpreted in terms of our model–natural variants in Hsp90 exhibit high robustness due to long-term stabilizing selection. De novo mutations from mutation accumulation studies in the laboratory, on the other hand, which have not been filtered through selection, can display a wide range of robustness levels. Similarly, a study of the yeast TDH3 promoter shows polymorphisms in natural populations have less expression noise than random mutations, indicating that natural selection maintains mutations with high robustness (Metzger et al., 2015). Our result that environmental change induces a selectively advantageous loss of robustness is supported by extensive studies over decades in bacteria, yeast and cancer cells (Galhardo et al., 2007, for review).

### 4.4 | Limitations

The use of Boolean models in this study has both advantages and limitations. The key advantage is computational simplicity in addressing otherwise unanswerable generic questions about evolution, a point first established in the groundbreaking and transformative work of Kauffman (1969), which established the capability of completely random genetic networks to self-organize and canalize. Later studies with such models explicitly treated the effects of fitness (Kauffman & Weinberger, 1989). In this work, we modelled populations of 10^4^ individuals, each containing 10^4^ genes controlling 10^3^ traits, which interact with an environment containing 10^3^ features over about 10^7^ generations. This population size is large or completely unreachable in terms of laboratory experiments on metazoa, and the number of generations modelled is equivalent to about 300 000 years in a *Drosophila* population with a 10-day generation time, or about 380 years for a bacterial chemostat experiment involving organisms that divide every 20 min. The price of such evolutionary realism in time scale and population size is a severe coarse-graining of the internal states of the model.

This coarse-graining of internal states means that we do not consider constraints imposed by the chemical and physical implementation of living systems. This failing is a property of Boolean models as a class. They are ill-suited to detailed comparison with experimental data, particularly at the molecular level. Such data almost never presents itself to the experimentalist in unambiguous terms of ZERO and ONE. Concomitantly, the components of Boolean models cannot be compared in an unambiguous and one-to-one manner with experimentally manipulable entities such as promoters, enhancers and enzymes. Given current limitations on both the fundamental understanding of biological systems and on available computing power, it is difficult to see how this limitation to generic evolutionary processes can be overcome in the near future.

In terms of establishing generic properties of multicellular evolution, we believe that the main limitation of this study is that it considers the effects of the hierarchical nature of organismal fitness arising from the interplay of many cellular states, but fails to consider the constraint imposed by the fact that multicellular organisms develop from a single cell over time. It has been suggested that a key mechanism of evolutionary change is alteration of the relative timing of developmental events, a phenomenon known as heterochrony (Gould, 1977). Addressing the effect of heterochrony on the relationship between canalization and evolvability requires incorporating ontogeny explicitly into a population genetics picture of evolution. Previous work has shown that generic models of the evolution of ontogeny have the potential to answer important questions about evolution, but computational obstacles remain (Mjolsness et al., 1995).

We did not explicitly consider the evolution of the genotype–phenotype map in this study, but we consider it a limitation less severe than the ones discussed above. Fixing the genotype–phenotype map was an *ansatz* used to clarify the role of the evolution of developmental control genes as distinct from those controlling basic cellular processes, and appears to be a necessary choice at the level of mechanistic resolution used here.

### 4.5 | Implications

The key ideas about canalization are all present in Waddington’s original short article (Waddington, 1942), a work whose intellectual fecundity is nothing less than astonishing. The dynamical metaphors in this article inspired Rene Thom to construct an entirely new branch of mathematics (Thom, 1969, 1975). Among evolutionary biologists, Waddington’s work was seen in terms of phenotypic robustness to genetic variation, the relief of which tended to increase evolvability (Félix & Barkoulas, 2015; Masel & Trotter, 2010, for reviews). This latter viewpoint found experimental support in the phenotypic properties of single cell organisms, protein and RNA structure, and models of single cell networks. And yet, what Waddington presented is an attempt to solve the seemingly Lamarckian phenomenon of adaptation to the environment by the non-Lamarckian mechanisms established by genetics. He did so specifically in terms of animal development, stating that ‘There seems, then, to be considerable amount of evidence from a number of sides that development is canalized in the naturally selected animal (Waddington, 1942, p. 564)’. Moreover, he argued that canalization itself led to adaptation in organisms which undergo development: ‘The particular application of this general thesis which we require in connexion with “the inheritance of acquired characters” is that a similar canalization will occur when natural selection favours some characteristic of which the environment plays an important part (Waddington, 1942, p. 564)’.

This tension between Waddington’s original idea that canalization is required for adaptation and the modern consensus that canalization must be relieved for adaptation to take place is resolved by the results presented in this article. On the one hand, we find all elements of the modern consensus present in the results considered here. High robustness leads to better fitness in a constant environment, and lower robustness leads to higher fitness during adaptation following an abrupt environmental change. At the same time, in multicellular organisms, the absolute levels of robustness are close to unity and ‘relief’ of robustness still involves values of R above 0.98. This contrasts sharply with the result of a non-multicellular model where optimal robustness ranged from 0.2 to 0.8 (Draghi et al., 2010). Resolving this apparent paradox required the use of a quantitative measure of robustness used with a model that explicitly represented multicellularity, phenotype and environment.

Although we believe that we have resolved an important issue arising from Waddington’s article, many important questions remain unresolved. A key issue is Waddington’s proposal that adaptation occurs by the genetic assimilation of traits that arise from phenotypic plasticity. Neither of these mechanisms were incorporated into the model considered here. Genetic assimilation was experimentally demonstrated in an artificial situation by Waddington (1953). Nevertheless, any form of adaptation, with or without phenotypic plasticity, can be viewed as a response to the environment. In natural situations, such as eye loss in the cavefish (Rohner et al., 2013), selection on phenotypic plasticity cannot be distinguished from selection on an increased range of genetically determined phenotypes produced by decanalization. One central unanswered question is the relative roles of these two processes over the course of evolutionary time.

More generally, the work reported here represents a small step towards resolving a theoretical tension at the heart of evolutionary biology. Evolutionary theory is formulated in terms of population genetics, in which the effects of genes are additive, with epistasis present in at most a small corrective term. In contrast, developmental genetics itself and consequently the theories describing it are entirely epistatic, with no additive behaviour at all. This tension has been noted by Erwin and Davidson (2009), although no theoretical solution was provided. Naturally, improved comparative developmental analyses of diverse organisms at the molecular level will be important. In addition, we believe that new mathematical ideas by theoreticians will be of great value.

## Supplementary Material

Jiang et al. 2023 Supplementary Information

## Figures and Tables

**TABLE 1:** List of variables.

Vector	Element	Element definition
v	$v_{j}$	*j*th genotype
z	$z_{i}$	*i*th phenotype
T	$t_{ij}$	Functional contribution of genotype j on trait i
h	$h_{i}$	Threshold for trait i
b	$b_{i}$	*i*th element in the environment, representing desired phenotype for $z_{i}$ under current environment

**TABLE 2:** List of parameters.

Parameters	Definition	Value(s)
c	Probability of $t_{ij}$ not 0	0.5
a	Proportion of 1s in v	0.5
K	Length of phenotype vector z	1000
L	Length of genotype vector v	10000
γ	Activation/repression coefficient of each gene on its target trait	0.1, 1, 10
α	A second parameter to scale robustness	0.5, 1, 2, 2.5, 3, 3.5, 4, 8, 16, 64, 128, 256
μ	Mutation rate per gene	10^−6^
N	Population size	10000
$\mu_{r}$	Mutation rate on robust genotype	10^−5^

## Data Availability

We have created a snapshot of our simulation code publicly available on Github and linked it with Zenodo (https://doi.org/10.5281/zenodo.7768897).
