# Supplementary material for: The effect of mutational robustness on the evolvability of multicellular organisms and eukaryotic cells
Source: J Evol Biol. Author manuscript; Available in PMC 2023 Jul 2. (PMC10315174; doi:10.1111/jeb.14180)
Supplement: Jiang et al. 2023 Supplementary Information [file NIHMS1906080-supplement-Jiang_et_al__2023_Supplementary_Information.pdf]

Supplementary materials

Table S1. Robustness values (R) for Figure S3

L \ γ	0.1	0.3	1	3	10
100	0.9960	0.9885	0.9719	0.9624	0.9606
1000	0.9987	0.9964	0.9911	0.9880	0.9875
10000	0.9996	0.9988	0.9972	0.9962	0.9960

Table S2. Robustness genotype and corresponding values of α

robustness genotype	α value
000	0.5
001	1
010	2
011	2.5
100	3
101	3.5
110	4
111	8

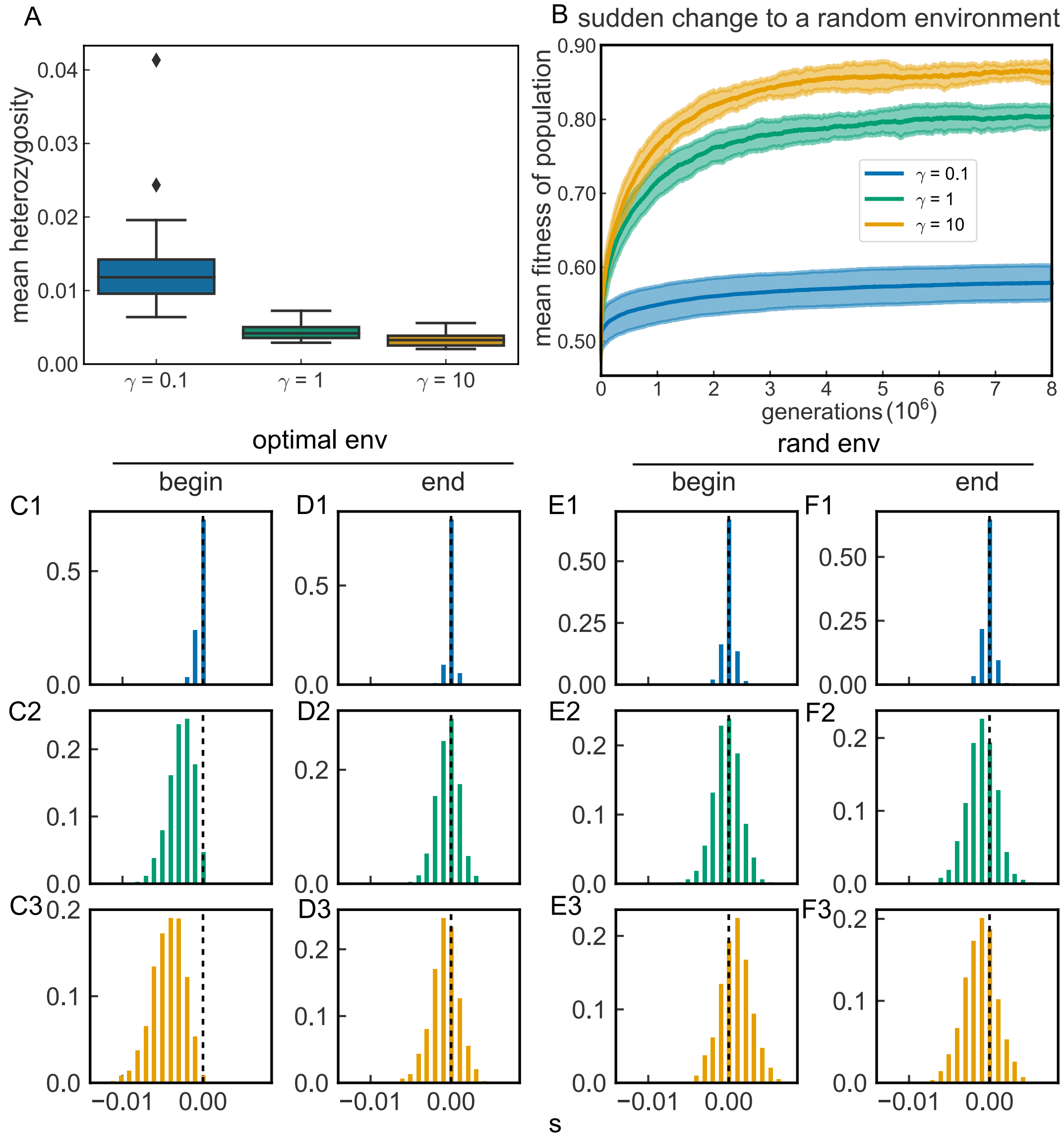

Figure S1. Population statistics when γ controls robustness. The same color scheme is used for γ as in the main text. Yellow: $\gamma = 0.1$, green: $\gamma = 1$, blue: $\gamma = 10$. A. Boxplot of heterozygosity at the end of simulations (8×10^6 generations) under E_{opt}. B. Mean fitness changes over time for populations that have undergone a sudden environmental shift (to E_{new}) which were previously in E_{opt} for 8×10^6 generations. C-F show the distribution of selection coefficient s in E_{opt} and E_{new} at different times. Dotted lines show the position of x=0. C, D are for populations in E_{opt}, with C at generation 0, and D at generation 8×10^6. E, F are for populations in E_{new}, with E at generation 0 and F at generation 8×10^6.

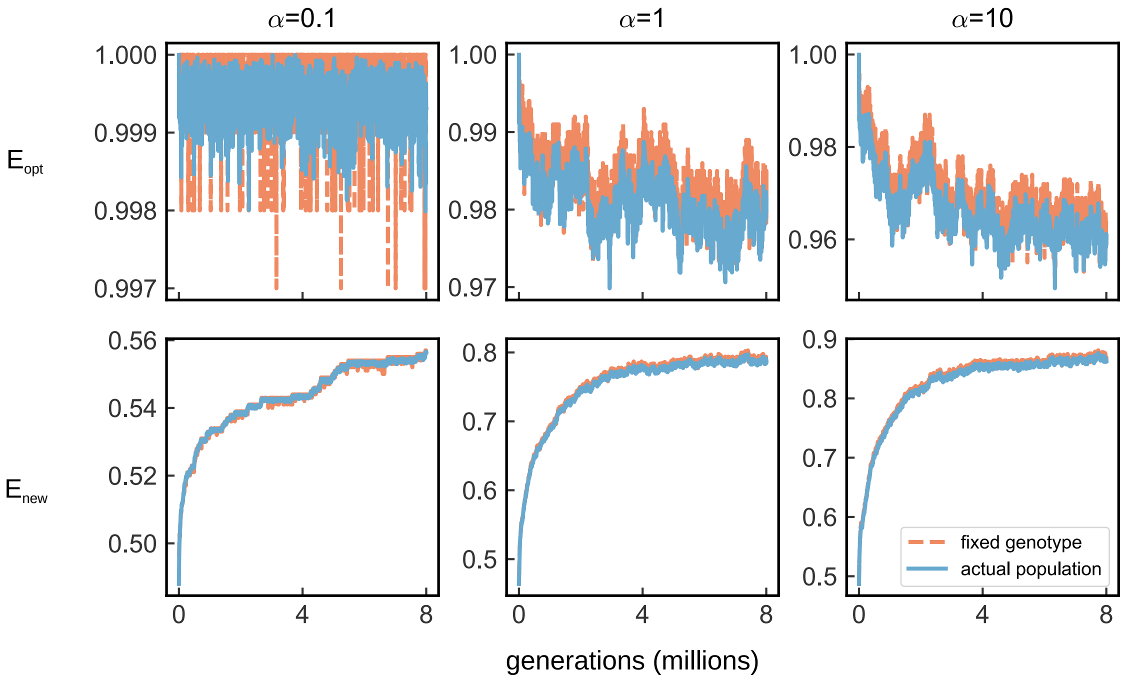

Figure S2. Population dynamics are driven by fixed mutations. Comparisons of the mean fitness of population over time versus the fitness of fixed alleles only. One example simulation is shown for each γ, for both E_{opt} and E_{new}.

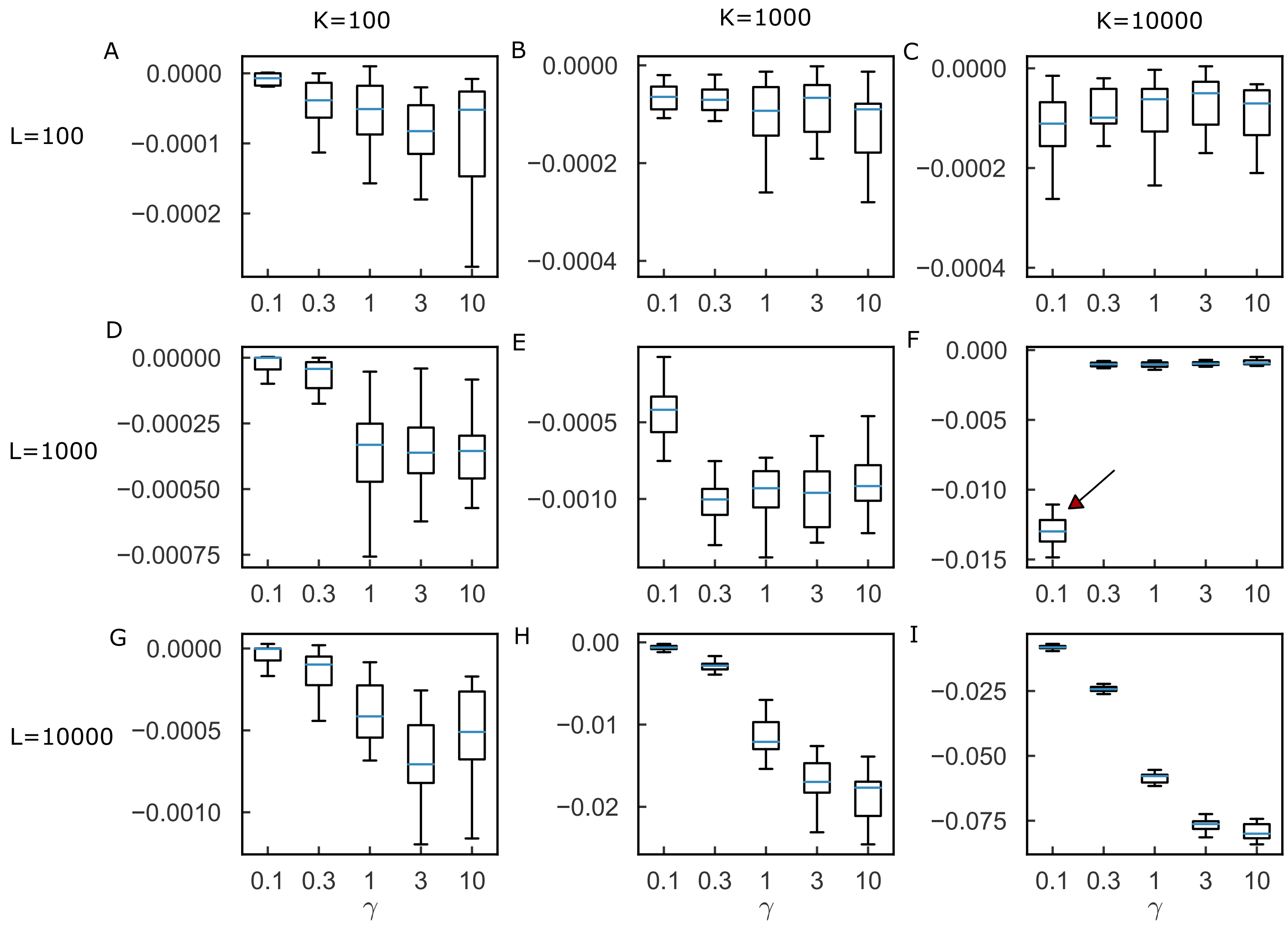

K=100
K=1000
K=10000
L=100
L=1000
L=10000
A
B
C
D
E
F
G
H
I
0.0000
−0.0001
−0.0002
−0.0004
0.00000
−0.00025
−0.00050
−0.00075
−0.0005
−0.0010
0.000
−0.005
−0.010
−0.015
0.00
−0.01
−0.02
−0.025
−0.050
−0.075
0.1
0.3
1
3
10
γ

Figure S3. Simulations under different parameter regimes. We varied K and L in the simulations, and in order to compare mean fitness differences, we set the simulations to run so that each simulation has the same expected number of mutations, so that for $L = 100$, simulations were run for 8×10^6 generations; $L = 1000$: 8×10^5 generations; $L = 10000$: 8×10^4 generations. Here we plot mean fitness differences. For $L = 10000$, as described in the main text, there is a monotonic relationship between robustness and the distance to optimal fitness. We observe that at $L = 1000$, the relationship of robustness to optimal fitness drastically shifts when K changes from 1000 to 10000 (arrow). When $K = 10000$ and $\gamma = 0.1$, the deleterious effects of individual mutations are sufficiently small that they get fixed by genetic drift, while for other γ values mutations have a larger effect on fitness and do not fix.

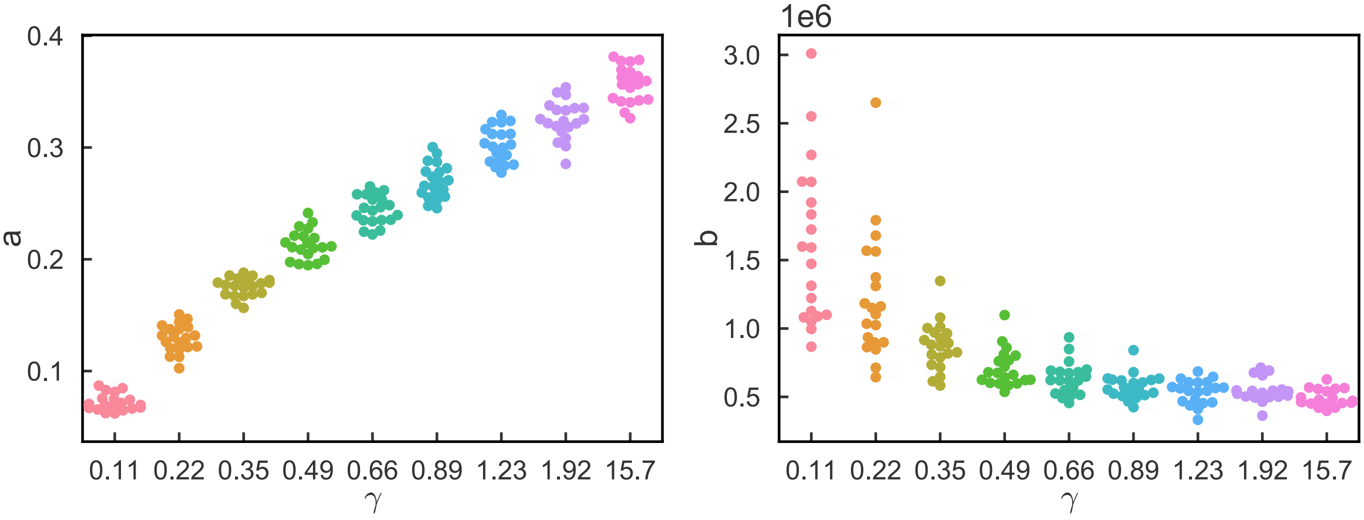

Figure S4. Monotonic relationship of robustness to adaptation rate with γ. To characterize the rate of adaptation for different robustness levels, we fit each mean fitness changes over time to the saturation function $y = at/(b + t) + c$ [Kirk, 2006]. a describes saturation level, while b describes the time needed to reach saturation. Here we plot the Durant distribution of a and b with populations that are equally separated in robustness.

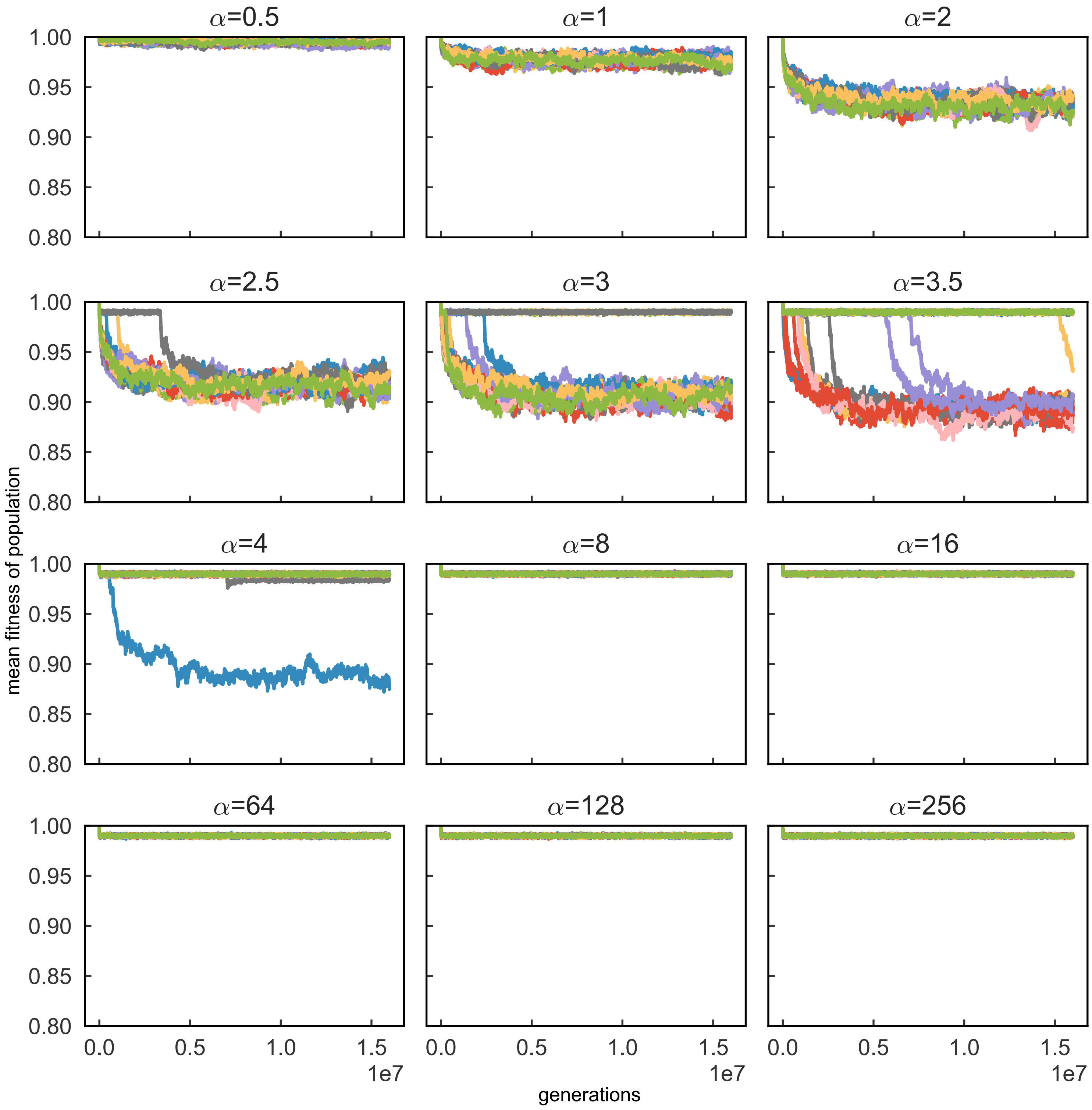

Figure S5. Examining the behaviors of alphas spanning its robustness range for a constant optimal environment. α values of 0.5, 1, 2, 3, 3.5, 4, 8, 16, 64, 128, 256 were examined.

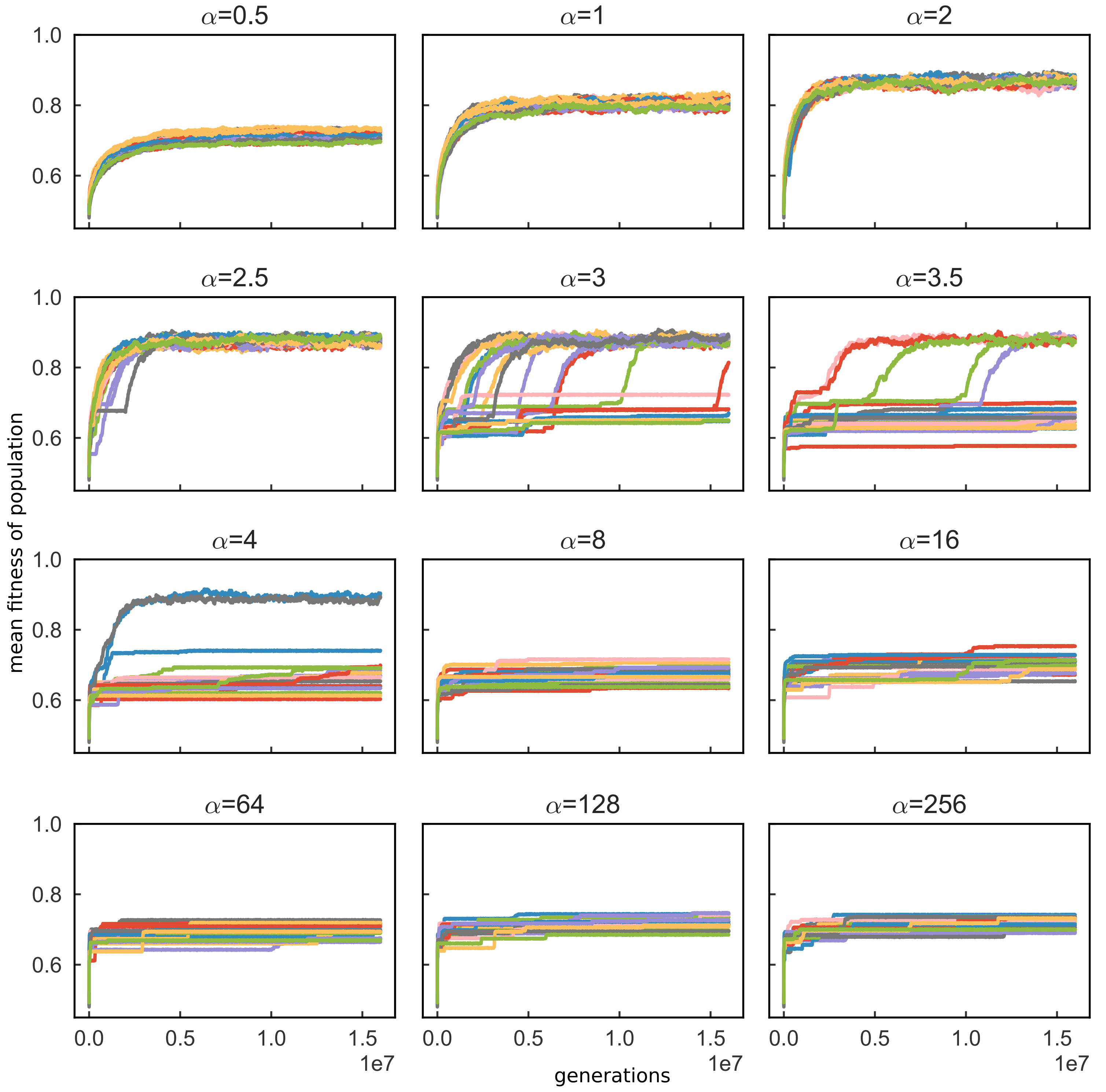

Figure S6. Examining the behaviors of alphas spanning its robustness range for adapting to a new random environment. α values of 0.5, 1, 2, 3, 3.5, 4, 8, 16, 64, 128, 256 were examined.

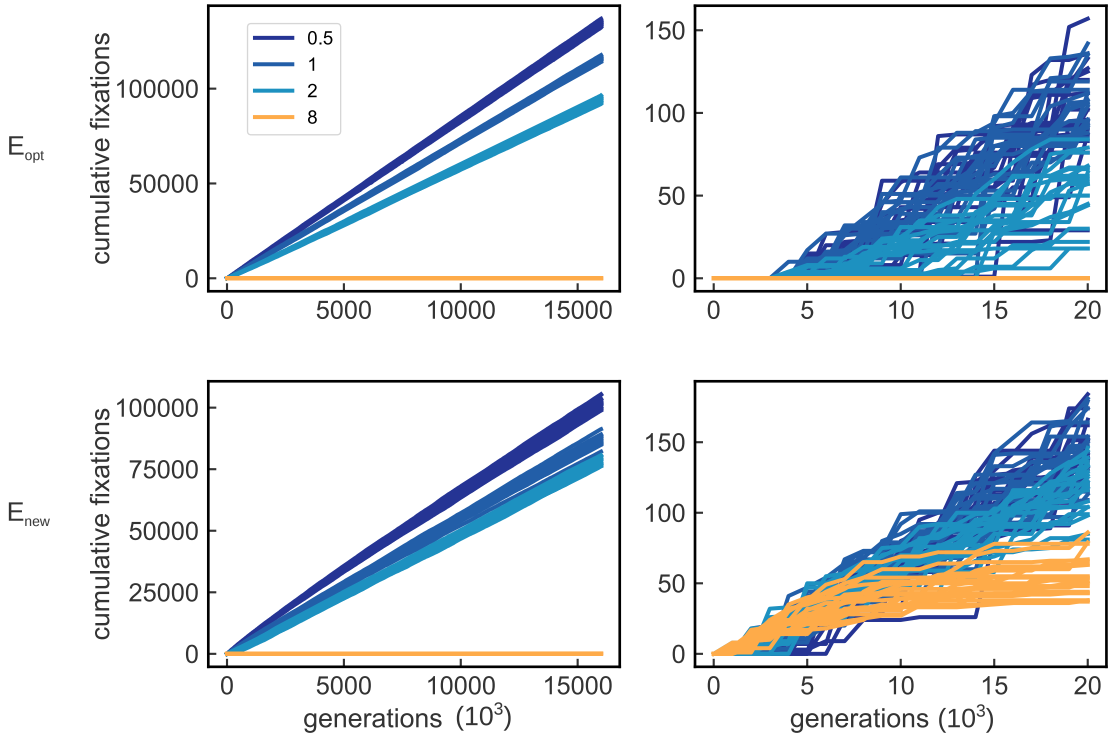

Figure S7. Cumulative number of fixations over time. Each plot shows the cumulative number of fixations in each of 20 simulations for each value of α.

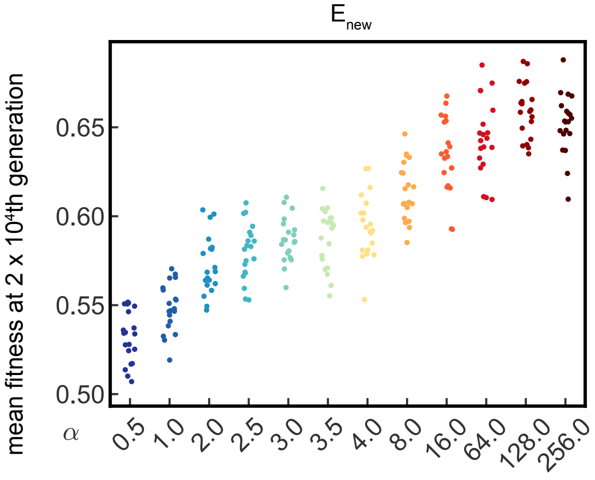

Figure S8. Mean fitness of population after the first 2×10^4 generations under $\mathrm{E_{new}}$

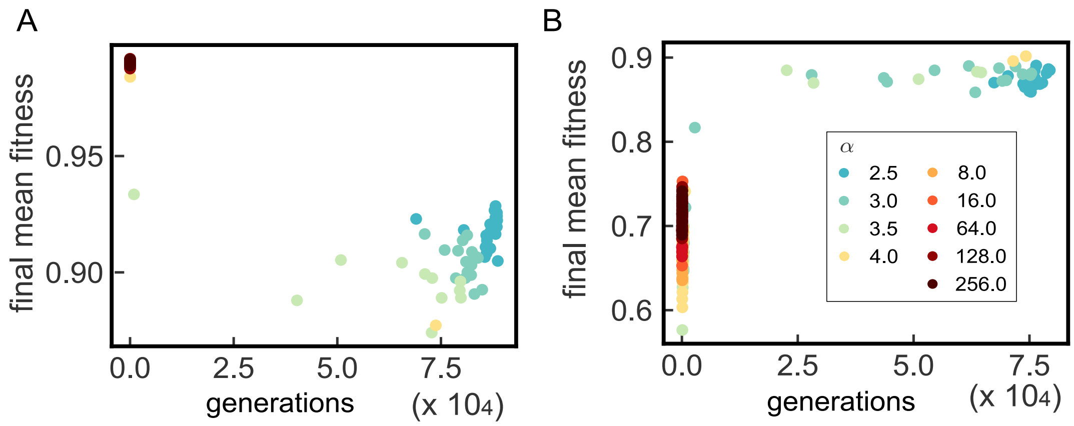

Figure S9. Cumulative number of fixations in relation to the final mean fitness. A. The relationship of the cumulative number of fixations to the final mean fitness of population under E_{opt}. Only for α values from 2.5 to 256. B. Same as A under E_{new}.

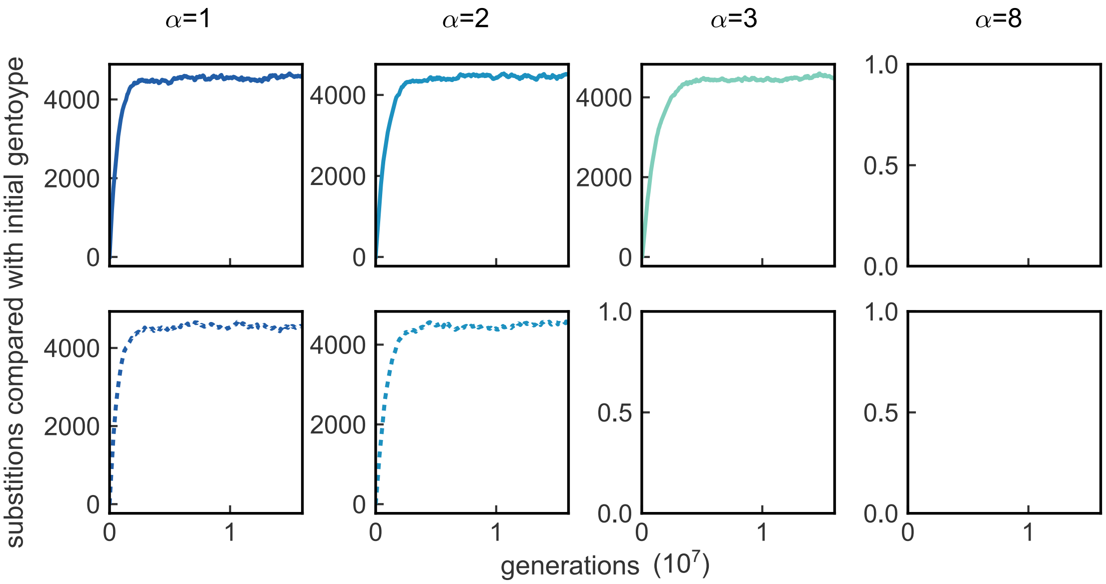

Figure S10. Substitutions under $\mathrm{E_{opt}}$**.** Here we show two simulations for α values 1, 2, 3 and 8. The same simulation was used as in Figure 5A. The solid and dashed lines in this figure correspond to the solid and dashed lines respectively in 5A. Absence of fixation after a particular time results in no line being plotted.Results from every 40,000 generations are shown.

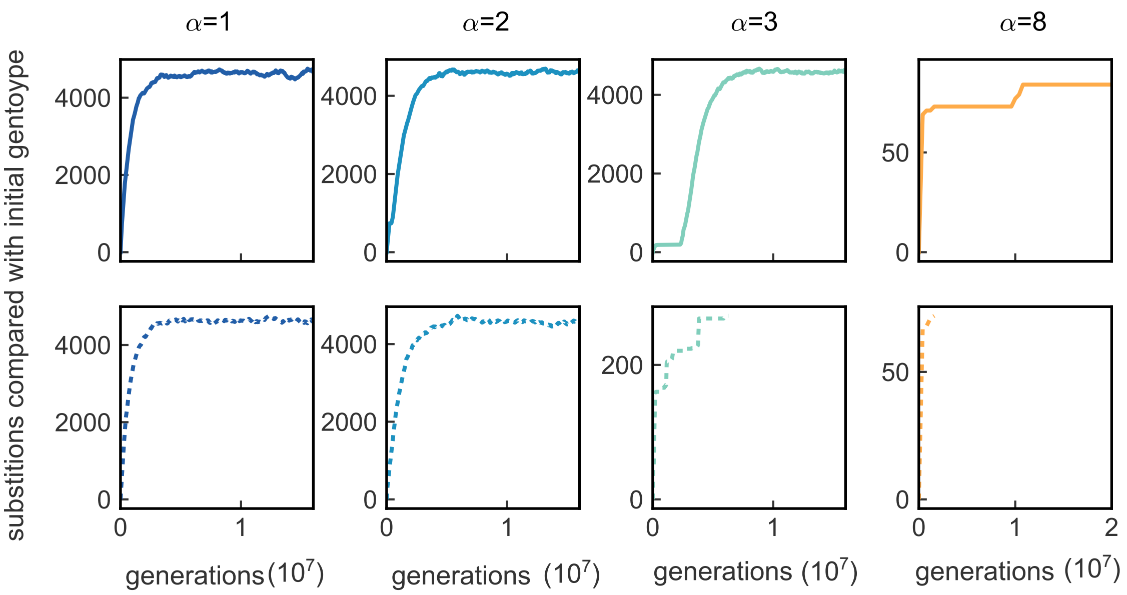

Figure S11. Substitutions compared to initial genotype under E_{new}**.** Here we show two simulations for α values 1, 2, 3 and 8. The same simulation was used as in Figure 5B. The solid and dashed lines in this figure correspond to the solid and dashed lines respectively in 5B. Absence of fixation after a particular time results in no line being plotted. Results from every 40,000 generations are shown.

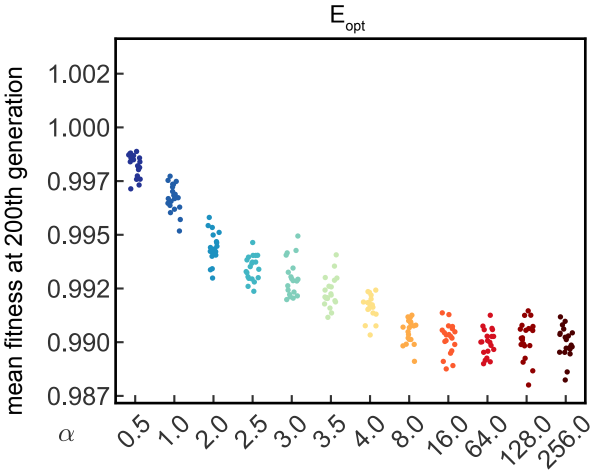

Figure S12. Mean fitness of population after the first 200 generations under $\mathrm{E_{opt}}$

References

David Kirk. Saturation curve analysis and quality control. *Shot Peener*, 20(3):24, 2006.
